# Clonal Expansion of Early to Mid-Life Mitochondrial DNA Point Mutations Drives Mitochondrial Dysfunction during Human Ageing

**DOI:** 10.1371/journal.pgen.1004620

**Published:** 2014-09-18

**Authors:** Laura C. Greaves, Marco Nooteboom, Joanna L. Elson, Helen A. L. Tuppen, Geoffrey A. Taylor, Daniel M. Commane, Ramesh P. Arasaradnam, Konstantin Khrapko, Robert W. Taylor, Thomas B. L. Kirkwood, John C. Mathers, Douglass M. Turnbull

**Affiliations:** 1 Newcastle University Centre for Brain Ageing and Vitality, Institute for Ageing and Health, Newcastle University, Newcastle upon Tyne, United Kingdom; 2 Wellcome Trust Centre for Mitochondrial Research, Institute for Ageing and Health, Newcastle University, Newcastle upon Tyne, United Kingdom; 3 Institute of Genetic Medicine, Newcastle University, Newcastle upon Tyne, United Kingdom; 4 Centre for Human Metabolomics, North-West University, Potchefstroom, South Africa; 5 Human Nutrition Research Centre, Institute for Ageing and Health, Newcastle University, Newcastle upon Tyne, United Kingdom; 6 Beth Israel Deaconess Medical Center, Boston, Massachusetts, United States of America; 7 Institute for Ageing and Health, Newcastle University, Campus for Ageing and Vitality, Newcastle upon Tyne, United Kingdom; Max Planck Institute for Biology of Ageing, Germany

## Abstract

Age-related decline in the integrity of mitochondria is an important contributor to the human ageing process. In a number of ageing stem cell populations, this decline in mitochondrial function is due to clonal expansion of individual mitochondrial DNA (mtDNA) point mutations within single cells. However the dynamics of this process and when these mtDNA mutations occur initially are poorly understood. Using human colorectal epithelium as an exemplar tissue with a well-defined stem cell population, we analysed samples from 207 healthy participants aged 17–78 years using a combination of techniques (Random Mutation Capture, Next Generation Sequencing and mitochondrial enzyme histochemistry), and show that: 1) non-pathogenic mtDNA mutations are present from early embryogenesis or may be transmitted through the germline, whereas pathogenic mtDNA mutations are detected in the somatic cells, providing evidence for purifying selection in humans, 2) pathogenic mtDNA mutations are present from early adulthood (<20 years of age), at both low levels and as clonal expansions, 3) low level mtDNA mutation frequency does not change significantly with age, suggesting that mtDNA mutation rate does not increase significantly with age, and 4) clonally expanded mtDNA mutations increase dramatically with age. These data confirm that clonal expansion of mtDNA mutations, some of which are generated very early in life, is the major driving force behind the mitochondrial dysfunction associated with ageing of the human colorectal epithelium.

## Author Summary

Mitochondrial DNA (mtDNA) mutations have been shown to accumulate with age in a number of human stem cell populations and cause mitochondrial dysfunction within individual cells resulting in a cellular energy deficit. The dynamics by which mtDNA mutations occur and accumulate within individual cells (known as clonal expansion) is poorly understood. In particular we do not know when in the life-course these mtDNA mutations occur. Here we have measured mtDNA mutation frequency using three different techniques; Random Mutation Capture, which measures low level mutation frequency as an indirect measure of mutation rate, Next Generation Sequencing, which measures clonally expanded mtDNA mutation frequency, and mitochondrial enzyme histochemistry as a marker of clonally expanded mtDNA mutations, on colorectal mucosal biopsies obtained from 207 healthy participants aged 17–78 years. We show that, by 17 years of age, there is a substantial mtDNA point mutation burden and that clonal expansion of early to mid-life mtDNA mutations is likely to be the cause of mitochondrial dysfunction associated with ageing in the human colon.

## Introduction

Mutations of mitochondrial DNA (mtDNA) have been implicated in the ageing process [1]. As humans age, multiple different mutations arise somatically in individual cells and some of these expand clonally to high levels over time, resulting in focal respiratory chain deficiencies [2]–[6]. To date there is poor understanding of the dynamics of mutation accumulation during ageing and of when in the life-course the majority of these clonally expanded somatic mtDNA mutations initially occur [7]. For example, there have been suggestions that the underlying mechanism involves either an accelerating mtDNA mutation rate over time [8], or clonal expansion of mtDNA mutations which have occurred in early life [7].

Resolving these questions about the dynamics of mtDNA point mutations is important because of their accumulation in human stem cell populations with age, which results in respiratory chain dysfunction [5], [9]–[14] and consequent reductions in cell function [15]. Age-related dysfunction of somatic stem cells has been proposed to lead to the decreased ability of tissues to regenerate [16]. Mice with increased mtDNA mutagenesis (mutator mice) caused by a defect in the proof-reading ability of the mitochondrial DNA polymerase gamma also show a premature ageing phenotype [17], [18] which has been attributed largely to somatic stem cell dysfunction resulting from high mtDNA point mutation loads [19]–[22]. In mutator mice the majority of the mutational burden leading to a cellular phenotype occurs during embryogenesis [19]. However, in human tissues there have been no comprehensive studies examining mtDNA point mutation occurrence and accumulation over the life-course.

Here, we apply different validated techniques to investigate the frequency of both low level (as an indirect measure of mutation rate) and clonally expanded mtDNA mutations, using the human colonic epithelium as an exemplar tissue with a well-characterised stem cell population. We found no evidence of a significant increase in the frequency of low level mtDNA mutations with age, but there was a significant increase in the frequency of clonally expanded mtDNA mutations with age. We provide robust evidence that mtDNA mutations occur early in life and that a substantial mtDNA point mutation burden exists within the human colorectal epithelium before the age of 20.

## Results

### No significant increase in low level mtDNA mutation frequency with age

**Figure 1:**
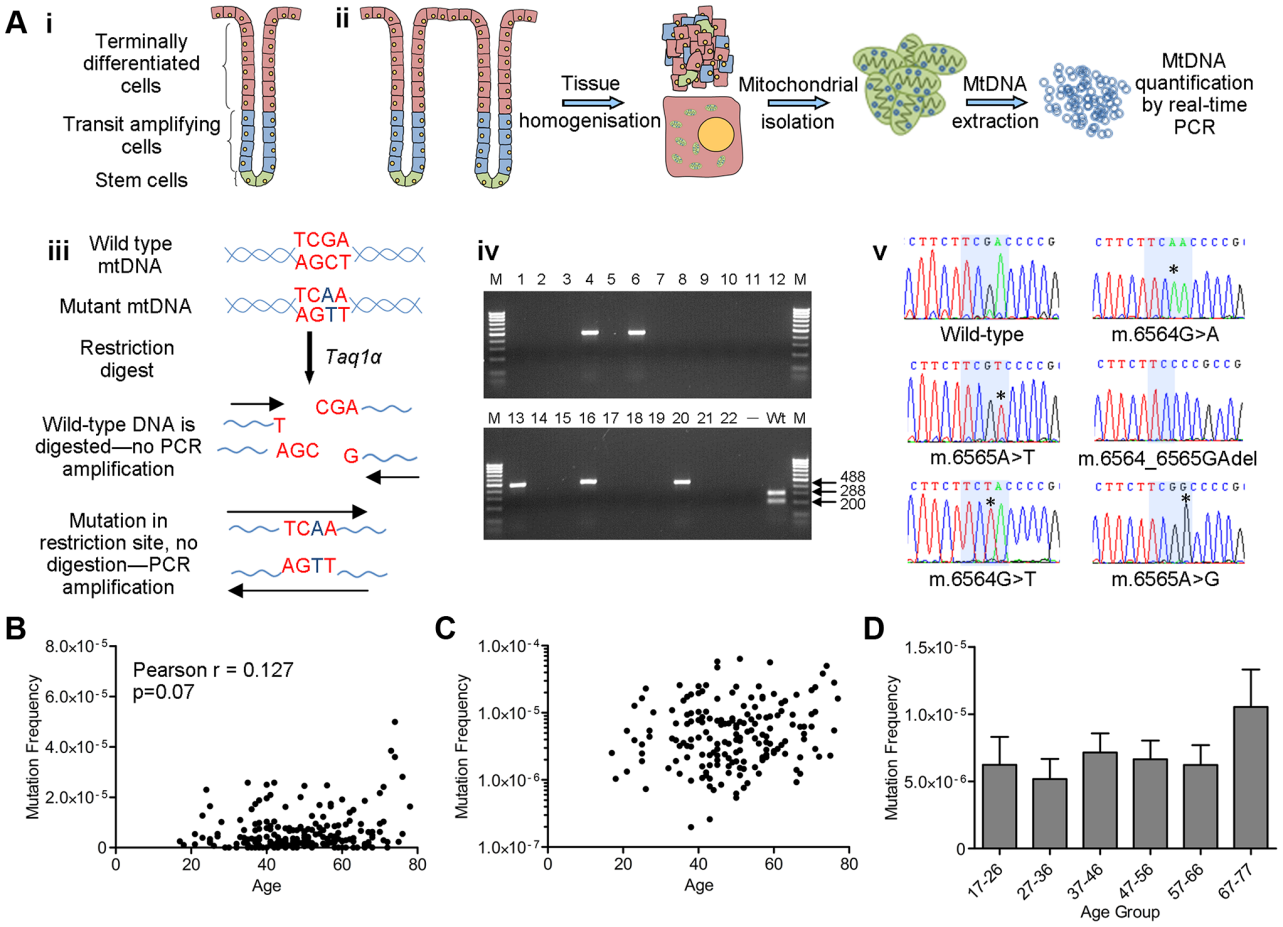
Analysis of mitochondrial DNA point mutation frequency with age by Random Mutation Capture (RMC). (**A**) Schematic diagram describing the RMC methodology. (**i**) Schematic diagram of the structure of the human colorectal crypt. (**ii**) Schematic diagram showing mtDNA isolation. Colonoscopic biopsies are homogenized and the mitochondrial fraction isolated by differential centrifugation. MtDNA is then prepared by phenol/chloroform extraction and quantified using real-time PCR (standard curve method). (**iii**) MtDNA is digested for 10 hours with *Taq1α*. PCR is then carried out over the restriction site. Only molecules with mutations in the restriction site are able to successfully amplify. (**iv**) Agarose gel showing PCR products from a typical RMC run. Each reaction contained ∼10000 target base pairs. 488 base pair bands show amplified mutated molecules (wells 4,6,13,16 and 20). The wild-type control well (Wt) shows complete digestion of wild-type DNA following PCR. (**v**) Example electropherograms showing mutations (asterisks) within the restriction site (highlighted in blue). (**B**) Frequency of all RMC detected mtDNA mutations in human colorectal mucosa (n = 207). There was no correlation between mtDNA mutation frequency and age (Pearson correlation = 0.127 (P = 0.07)). (**C**) Data from (**B**) presented on a log 10 scale to show the spread of the data. Note that the zero values cannot be displayed in this way therefore n = 175. (**D**) Frequency of all mtDNA mutations detected in human colonic mucosa, grouped by decade. Subjects were grouped as follows, 17–26 (n = 12), 27–36 (n = 19), 37–46 (n = 58), 47–56 (n = 51), 57–66 (n = 43), 67–77 (n = 23). There were no significant differences between any of the groups (P = 0.343, One Way ANOVA).

Colorectal biopsies from 207 subjects aged 17–78 years with no evidence of bowel pathology at endoscopy were collected. Low level mtDNA mutation frequencies were quantified in these biopsy samples using a highly sensitive Random Mutation Capture (RMC) assay [23], [24] (Figure 1A). Approximately 200 million base pairs of mtDNA sequence were screened and a total of 803 mutations were detected. All mutations and full details of the number of bases investigated per individual are shown in Table S1. Examination of the types of mutational events detected by RMC in our cohort showed that 60% of all mtDNA mutations were G>A or C>T transitions, 24% were T>C or A>G transitions, and the remainder were transversions and small insertions and deletions. We also noted an uneven distribution of mutations across the four base pair TCGA *Taq*Iα site; 63% of changes were at the third base pair, with the remaining 37% spread fairly evenly across the other 3 bases. There was no significant correlation between low level mtDNA mutation frequency and age (Pearson correlation = 0.127 (p = 0.07, Figures 1B and 1C)). The RMC assay can be intrinsically noisy due to random sampling statistics [25], with inter-individual variation often observed in studies of ageing populations [26]. Therefore to maximise our chance of detecting a relationship between low level mtDNA mutation frequency and age, we pooled the data by decade of participant age. Although there was a modest increase in low-level mutation frequency with age (Figure 1D), this was not statistically significant (p = 0.343, One Way ANOVA). A Tukey post-hoc comparison revealed no significant differences even for the comparison between the first and the last decade of participant age. In a number of subjects we were not able to detect any mtDNA mutations in the base pairs screened. To ensure that these zero values were not having a significant effect on the data we re-ran the analyses excluding the zero values. There was still no significant association with age in either the individual data points (p = 0.07, Pearson correlation = 0.136) or the grouped data (p = 0.46, One Way ANOVA) (Figure S1). These data highlight that even the youngest person studied (aged 17 years) had an appreciable mtDNA mutation frequency of 2.5 mutations per 10^6^ base pairs. Collectively these analyses demonstrate no significant increase in low level mtDNA mutation frequency with age. There was no significant difference in the frequency of synonymous and non-synonymous mtDNA mutations in older (>46 years) vs younger participants (<46 years) (p = 0.665, Fisher's exact test). This confirms that there are no selective pressures acting on mtDNA point mutation occurrence with age and that mtDNA mutations present from early adulthood in the human colon could be pathogenic later in life if they were to clonally expand to high levels over time.

### Significant increase in the frequency of clonal expansions and low level heteroplasmic mtDNA mutations with age

**Figure 2:**
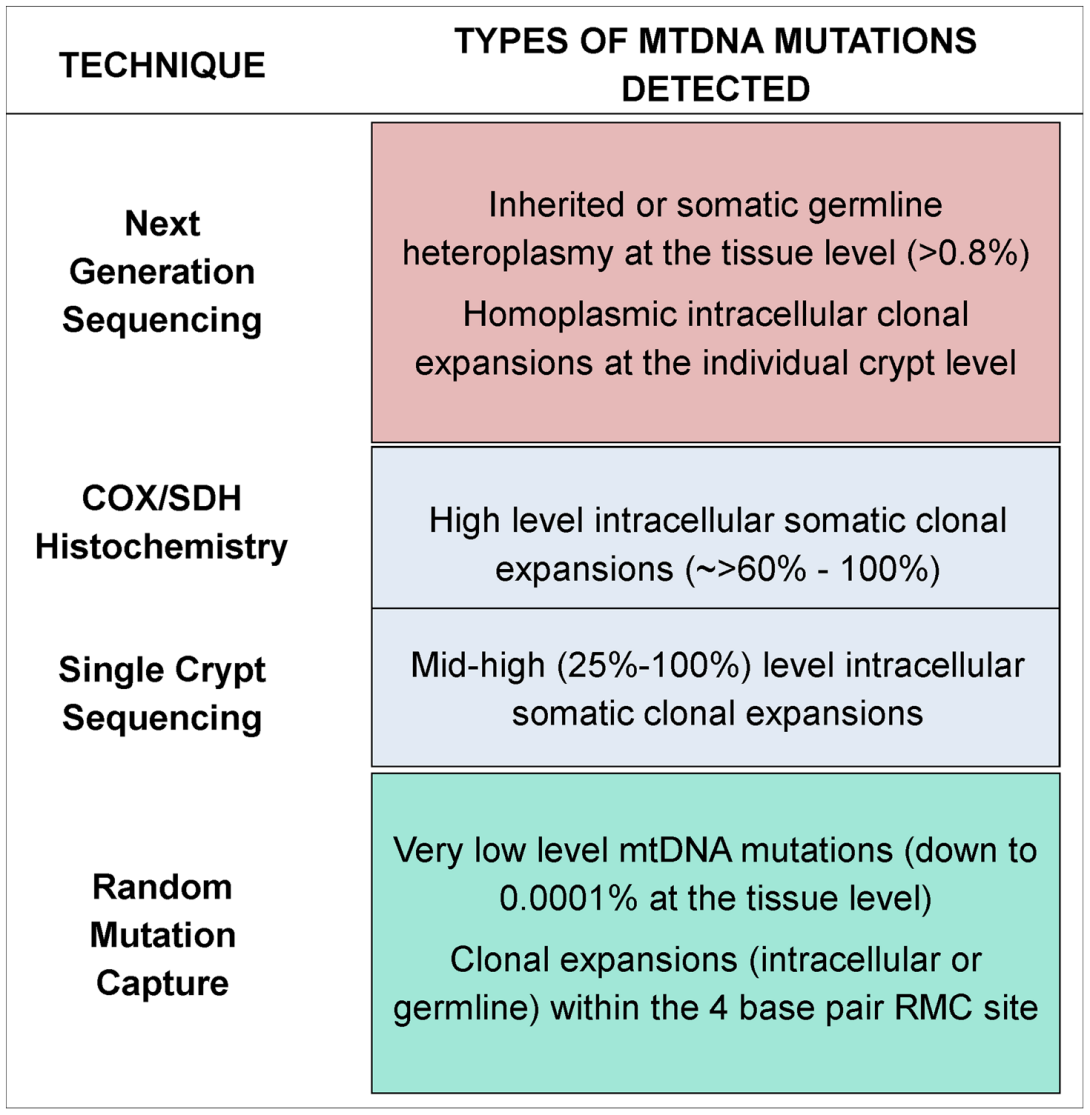
Schematic diagram showing the detection limits of each of the techniques utilised in this study.

Due to the small size of the region of the genome under investigation by the RMC assay, and the relative rarity of clonally expanded mutations across the entire mtDNA molecule, the RMC will not detect most of the clonally expanded mtDNA mutations. Indeed, in our RMC mutation dataset there were no mutations expanded to more than the equivalent of 1/100 of a crypt size. Therefore, we employed a next generation sequencing (NGS) approach to examine all mtDNA sites and gain information about the age dependent dynamics of clonally expanded mutations. Whole mtDNA Ion Torrent NGS was carried out on DNA extracted from the biopsies from a representative subset of the youngest (<26 years of age n = 8) and oldest participants (>70 years of age, n = 8) investigated by RMC. Our stringent quality control criteria (see Materials and Methods) set the heteroplasmy threshold for calling mtDNA mutations at 0.8%. Based on this figure and the average number of crypts per biopsy (∼200), our NGS assay could detect homoplasmic clonal expansions within individual crypts or clusters of clonal crypts [14], as well as low levels of heteroplasmic mtDNA mutations (>0.8%) present throughout the whole tissue [27], [28]. Figure 2 details the various techniques employed throughout the study and the limits of detection for each technique.

**Figure 3:**
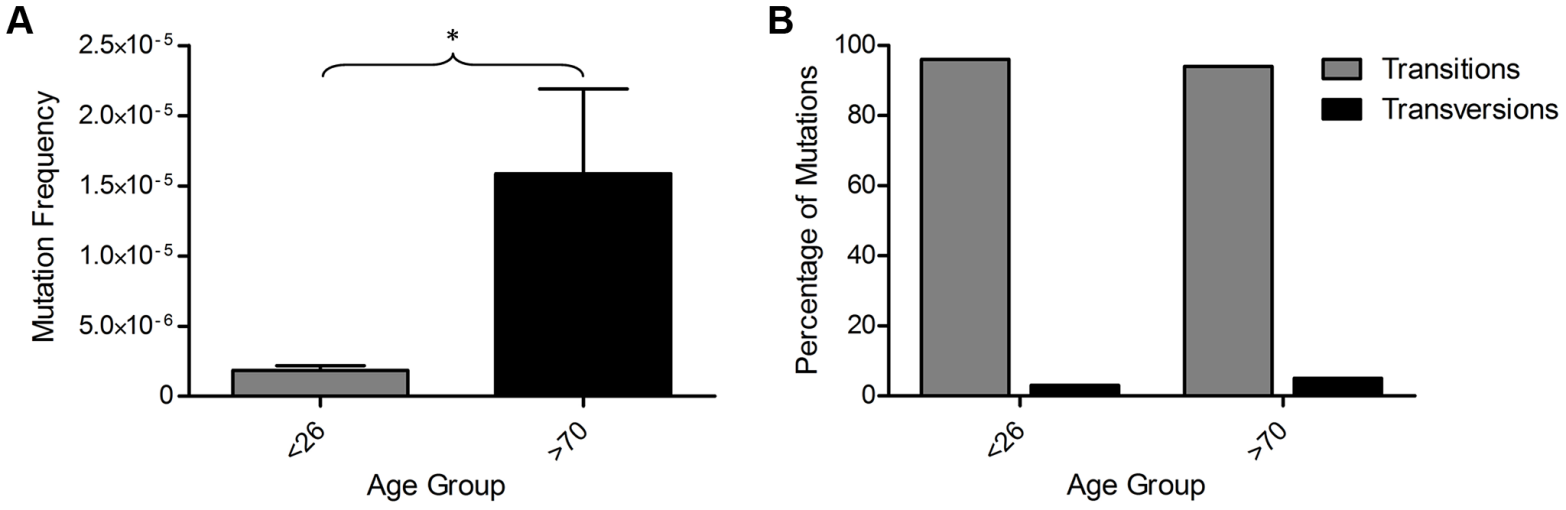
Mitochondrial DNA mutations in human colorectal epithelium of subjects below 26 years of age (n = 8) and over 70 years of age (n = 8) measured by Next Generation Sequencing (NGS). (**A**) MtDNA mutation frequency. There was a significantly higher mutation frequency in the subjects >70 years (p = 0.0361, unpaired t-test). (**B**) Types of mutations detected by NGS frequency. There was no significant difference in the types of mutations detected in subjects <26 and >70 years of age (p = 1.00, Fisher's exact test).

We sequenced a total of 556 Mb of mtDNA (average of 35 Mb per subject) and detected 109 mutations present at >0.8% heteroplasmy. All detected mtDNA variants are detailed in Table S2. All participants showed some variants at >0.8% heteroplasmy, but the frequency of mtDNA mutations was more than 8-fold higher in the older than in the younger group (Figure 3A, p = 0.036, unpaired t-test). There was no significant difference in the types (transitions/transversions) of mutations observed between the two age groups (Figure 3B, p = 1.00, Fisher's exact test), with single nucleotide transitions being by far the major mutation type.

A recent study has shown that recurrent tissue-specific mtDNA mutations are present in unrelated individuals [29]. We investigated our NGS data to determine whether recurrent mtDNA mutations were also present within our dataset. In our colon data we detected eight unique mtDNA mutations that were present in two individuals; however unlike the data from Samuels et al, they were not restricted to the non-coding regions, but appeared to be located randomly; three were in the non-coding region, three were in protein encoding genes and two were in RNA genes. Five of these were previously reported polymorphic variants [30], the remaining three were previously unreported. There is no evidence of contamination as they do not fit a haplogroup specific pattern with multiple markers seen in individual subjects [31], they were present in random pairs of subjects, and they were not observed in the yeast plasmid control.

### Exclusion of low level inherited heteroplasmic mtDNA mutations

It has recently been shown that mtDNA mutations in adult tissues can originate in embryonic development or even in the germline [27], [28], inferring that some of the mutations that we detected by NGS could have occurred during this period. To investigate this possibility, matched buccal scrape samples were collected at the same time as the colorectal mucosal biopsies from the same 16 participants from whom we had carried out NGS (data in Figure 3), and NGS was performed on DNA from the buccal cells. Identification of the same mtDNA mutations in two different tissues would support the hypothesis that such mutations occurred prior to tissue differentiation during embryogenesis. It should be noted that buccal and colonic epithelial cells both arise from the endoderm with the fore and hind gut becoming separate tissues by weeks 3–4 of gestation [32]. In DNA from the 16 participants investigated, we detected a total of 16 mtDNA mutations that were present at low levels of heteroplasmy in both tissues (Table S2 and Table S3) and these occurred in 10 participants. Five of these people were in the >70 year age group, five were in the <26 year age group. There was no significant difference in the frequency of germline or embryological mtDNA mutations between the <26 year and >70 year age group (p = 0.176, unpaired t-test) confirming that there was no age-effect and that these mtDNA mutations were most likely of germline or embryological origin.

**Figure 4:**
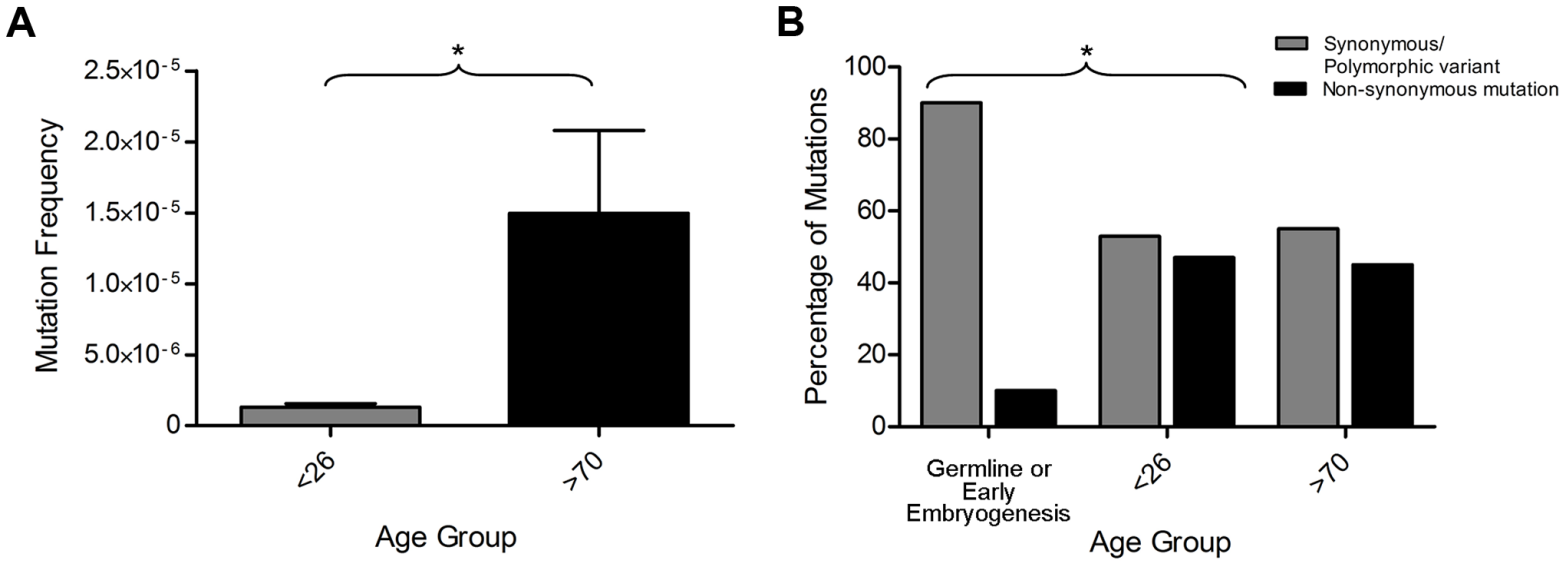
Exclusion of mitochondrial DNA (mtDNA) mutations occurring in the germline or in early embryogenesis. (**A**) Somatic mtDNA frequency (mtDNA mutations present in colon only) measured by NGS. There was a significantly higher mutation frequency in the subjects >70 years (P = 0.0351, unpaired t-test). (**B**) Percentage of synonymous/polymorphic variants and non-synonymous mtDNA mutations which were of germline or early embryological origin compared to those which were somatic in adults <26 years of age and over 70 years of age. There was a significantly lower frequency of non-synonymous mtDNA mutations in the somatic mtDNA mutation groups compared to the germline or early embryological mtDNA mutation group (p = 0.041 Fisher's, exact test).

The frequency of somatic mtDNA mutations in the colon samples was then analysed by subtracting the germline or embryological mtDNA mutations from the total mtDNA mutation frequency. This revealed a significant 10-fold increase in the frequency of clonally expanded somatic mtDNA mutations in those aged >70 years compared with <26 years (Figure 4A, p = 0.035, unpaired t-test). From here on the mtDNA mutations detected in colorectal epithelium only will be referred to as somatic mtDNA mutations and those present in both buccal and colorectal epithelium as germline or early embryological mtDNA mutations. The pattern of somatic mtDNA mutations detected in the buccal epithelium was similar to those in the colonic epithelium. The mtDNA mutations detected were base transitions and were randomly located throughout the genome. We did observe a higher number of somatic mtDNA mutations in the colonic epithelial samples compared to the buccal samples. We have previously shown that there are tissue specific differences in the frequency of clonally expanded mtDNA mutations, with the colon being one of the most highly affected [10] and believe that this could explain these differences.

Next we compared the ratio of synonymous or polymorphic protein encoding mtDNA mutations to non-synonymous mtDNA mutations in the somatic and germline or early embryological data sets to see if there were any differences between the two, and therefore any evidence for purifying selection. There was a significantly higher proportion of non-synonymous mtDNA mutations in the somatic data set compared with the germline or early embryological data set (Fisher's exact test with, p = 0.041, Figure 4B); in fact only one of the germline or early embryological mtDNA mutations was non-synonymous and therefore potentially pathogenic. These data suggest that the mtDNA mutations likely to contribute to the mitochondrial ageing phenotype begin to occur sometime after 3–4 weeks gestation (1–2 weeks post-conception), which coincides with the resumption of mtDNA replication which is thought to occur post-embryo implantation [33].

### A significant increase in the frequency of respiratory chain deficient colonic crypts with age correlates with the frequency of mutations detected by NGS

**Figure 5:**
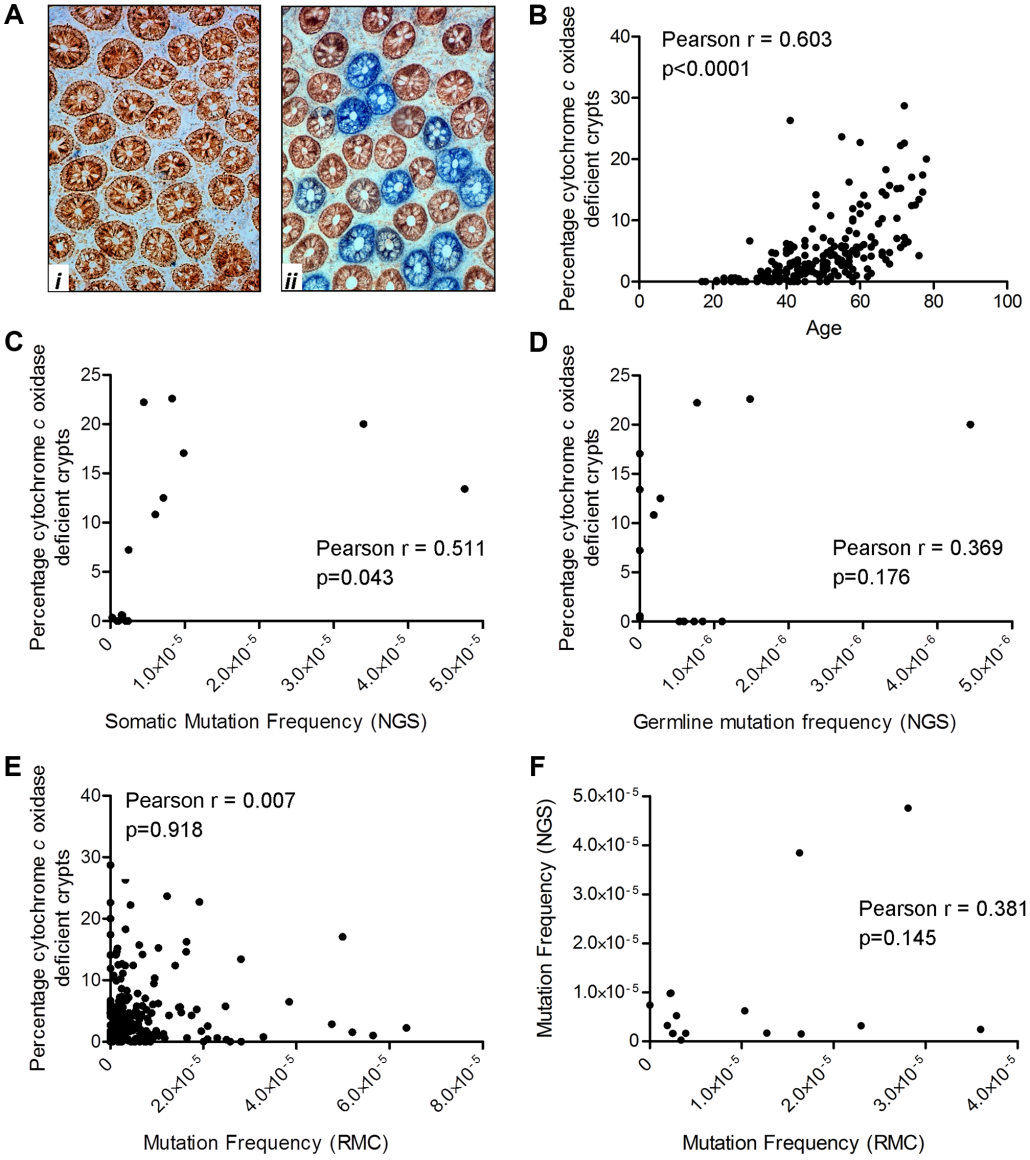
Quantification of mitochondrial dysfunction in human colonic crypts by cytochrome *c* oxidase (COX)/succinate dehydrogenase (SDH) histochemistry. (**A**) COX/SDH sequential histochemistry on a transverse section of human colorectal mucosa. The crypts which stain brown have functional COX and SDH activity; those which are blue have lost COX activity but retain SDH activity. (***i***) shows an image from a subject aged 25 in whom no COX deficient crypts were detected. (***ii***) shows an image from a subject aged 72 in whom 28% of crypts were COX deficient (**B**) COX activity was measured in colorectal biopsies from 207 subjects. A crypt was defined as deficient if more than 50% of cells had lost COX activity. There was a significant correlation between age and the percentage of COX deficient crypts. (Pearson correlation = 0.603, p<0.0001). (**C**) Correlation analysis between somatic mtDNA mutation frequency determined by NGS and percentage COX deficient crypts showed a significant correlation (Pearson correlation = 0.511, p = 0.043). (**D**) Correlation analysis between germline mtDNA mutation frequency determined by NGS and percentage COX deficient crypts showed no significant correlation (Pearson correlation = 0.369, p = 0.176). (**E**) Correlation analysis between the frequency of random mtDNA mutations by RMC and percentage COX deficient crypts showed no significant correlation (Pearson correlation = 0.007, p = 0.918). (**F**) Correlation analysis between the frequency of random mtDNA mutations by RMC and those by NGS in paired samples showed no significant correlation (Pearson correlation = 0.145, p = 0.381).

We have previously shown that the frequency of crypts deficient in cytochrome *c* oxidase activity (complex IV of the respiratory chain) increases with age in the apparently normal mucosa taken from patients with a colorectal tumour [5]. We also demonstrated that in the vast majority of these crypts there is an intracellular clonally expanded mtDNA point mutation [5], [34]. The present study provided an opportunity to determine whether there was a similar age-related increase in the frequency of crypts deficient in cytochrome *c* oxidase activity in healthy participants in whom there was no evidence of mucosal dysplasia. In addition, as cytochrome *c* oxidase (COX)/succinate dehydrogenase (SDH) histochemistry is an excellent surrogate marker for mid-high level intracellular clonally expanded mtDNA point mutations, which both RMC and NGS are quite likely to miss, this assay gives an indication of the frequency of such mutations (Figure 2). Colorectal mucosal biopsies collected by endoscopy from the same 207 subjects investigated by the RMC assay were subjected to sequential COX/SDH histochemistry (Figure 5A) and the percentage of COX deficient colonic crypts calculated. As expected, there was a significant increase in the percentage of COX deficient crypts in individuals with age (Figure 5B, Pearson correlation 0.603 (p<0.001)). The somatic mtDNA mutations detected using the Ion Torrent NGS platform could be clonally expanded mtDNA mutations in individual colonic crypts or low level clonally expanded mtDNA mutations present throughout the whole tissue. Therefore we investigated a possible correlation between the percentage of COX deficient crypts (known to be a good marker of clonally expanded mtDNA point mutations [5]) and the somatic mtDNA mutation frequency measured by NGS. This showed that there was a significant correlation between mtDNA mutation frequency as measured by NGS and COX deficient crypts (Figure 5C, Pearson correlation = 0.511 (p = 0.043)). When we compared the germline mtDNA mutation frequency with the percentage of COX deficient crypts, there was no significant correlation (Figure 5D, Pearson correlation = 0.369, p = 0.176). There was no significant correlation between the mtDNA mutation frequency detected by RMC and COX deficiency (Figure 5E, Pearson correlation = 0.007, p = 0.918), suggesting that the majority of pathogenic mtDNA mutations detected by NGS are somatic clonally expanded variants. In addition when we compared the RMC and NGS data from the same subjects side by side, there was no significant correlation confirming that the two assays were measuring different classes of mtDNA mutations i.e. low level vs clonally expanded (Figure 5F, Pearson correlation = 0.381, p = 0.145, Figure 2).

## Discussion

In this study we have examined the timing of occurrence and frequency of mtDNA mutations during ageing in human colorectal epithelium. We have employed a range of methodologies to provide accurate assessment of low level mtDNA mutation frequency, germline mtDNA heteroplasmy, and both high and low level intracellular clonal expansion. For low level mtDNA mutation frequency, we have shown previously that the most accurate method to use is the RMC assay [25], which provides the best available measure of mtDNA mutation rate [23]. An advantage of this assay is that it is not affected by false positive mutational calls caused by either PCR or sequencing errors; however a limitation is that, because only a small mtDNA domain is interrogated, it will miss the majority if not all intracellular clonally expanded mtDNA mutations. The NGS approach we have used is, in our hands, sensitive down to 0.8% heteroplasmy which correlates to either low-level germline mtDNA heteroplasmy or homoplasmic intracellular mtDNA clonal expansions in one or more crypts [14] (based on there being ∼200 crypts in a colonoscopic biopsy sample). Sequencing of individual laser micro-dissected crypts, which we have done extensively in our previous work [5], [34], is required to detect the remaining mtDNA mutations, i.e. mid-high level intracellular clonal expansions; these can also be detected using COX/SDH histochemistry as a surrogate marker [5], [14], [34], [35] as we have done here (Figure 2). There is the possibility that there are effectors of COX deficiency other than clonally expanded mtDNA point mutations, such as nuclear DNA mutations or changes in global gene expression; however we have previously found evidence of a clonally expanded mtDNA point mutation in >75% of crypts sequenced [5], [14], [34], [35] and therefore believe that this is an excellent surrogate marker. We have not previously detected any large-scale mtDNA deletions in individual COX deficient crypts [5].

The RMC assay revealed that there was no significant increase in low level mtDNA mutation frequency with age. This is supported by a published study which used RMC to measure mtDNA mutation frequency in colonic epithelium from a much smaller cohort of individuals covering a narrower age range (∼50–90 years of age (n = 20)) [23]. An important result of our study is a demonstration that the lack of an increase in low level RMC-detected mtDNA mutations (i.e. no increase in mtDNA mutation rate) does not mean that there is no increase in total mtDNA mutation load with age. Instead, we show that clonally expanded mutations, as measured by NGS, increase very dramatically. This is the first demonstration that mtDNA mutation rate and clonal expansion may follow very different age dynamics. This is in direct contradiction of the mitochondrial vicious cycle hypothesis of ageing [36] which suggests that mtDNA mutations occur during ageing leading to dysfunctional proteins in the oxidative phosphorylation system, precipitating increased mutation i.e. an accelerating mtDNA mutation rate over time. Our results also demonstrate that caution should be exercised in interpreting mutation analysis results, which may be limited to only a portion of mutations depending on the technique used.

Together the NGS and RMC datasets suggest that mtDNA mutation rate does not change significantly with age, but that clonal expansion of mtDNA mutations occurs over time. Mathematical modelling studies have suggested that clonal expansion of mtDNA mutations within an individual cell is likely to be due to random genetic drift and predict that it can take at least 20 years for an mtDNA mutation to clonally expand to high levels sufficient to cause COX deficiency [37]–[39]. Indeed, the youngest participant in this cohort in whom COX deficiency was detected was 21 years of age, therefore the initial mutational event(s) in this case must have occurred very early in life. Interestingly, the mtDNA mutation frequency data by NGS from our human samples were different to those obtained from a similar NGS study carried out in mice in which no significant increase in mtDNA mutation frequency with age was noted [40]. Previously we examined colonic epithelial tissue from a similar ageing mouse colony and showed that clonal expansion of mtDNA mutations was a very rare event in these animals compared with aged humans [41]. This may explain the species differences in these data, consistent with modelling studies that emphasise the difficulty of generating clonal expansion through random drift in short-lived animals [39].

Whilst our data imply that mutations of very early origin contribute to mitochondrial dysfunction in old age, it does not mean that mutations occurring in adult life play no role. In fact, the number of different clonally expanded mutations per sample detected by NGS robustly increased with age (3-fold from <26 years to >70 years group, p = 0.001). Such an increase in mtDNA point mutation diversity can be explained with a scenario whereby *de novo* mtDNA point mutations occurring during adult life, perhaps up to middle-age, are able to clonally expand and join the set of expanded mutations detected in old age. However mtDNA mutations which occur late in life will not have time to expand to high levels.

Analysis of the RMC data showed that even in the youngest participants, we observed a substantial load of mtDNA mutations in the colonic epithelium. NGS analysis of a subset of our youngest participants, all of whom were <26 years of age (n = 8), confirmed the RMC data; young adults have a significant mtDNA point mutation load. This has been previously shown to be the case in DNA extracted from young brain [42], [43], where both point mutations and mtDNA deletions have been detected. Our RMC data have now shown that this is also the case for a mitotic tissue, the colonic epithelium. In addition we have clearly demonstrated that the same type of mtDNA mutations (point mutations which are predominantly transitions) are present in young individuals as those detected in our previous studies of clonally expanded mtDNA mutations from aged respiratory chain deficient individual crypts [34], i.e. the seed mutations for clonal expansion can be laid down at an early age. Although this has been predicted by modelling simulations [37], [38] this is the first experimental evidence to show this definitively.

Our NGS analysis has shown that low level heteroplasmic mutations are present in multiple tissues from the same individual. This supports previous studies showing that mtDNA mutations in adult tissues can originate in germline or very early development [27], [28]. Indeed, our data from the colonic and buccal epithelium show that mtDNA mutations present in both tissues must have occurred prior to the fore and hind guts becoming separate which is thought to occur 1–2 weeks post-conception [32]. It is possible that due to our conservative cut off of 0.8% heteroplasmy, there may be additional germline mtDNA mutations which have undergone less drastic clonal expansion in one of the two tissues studied than the other, and therefore are below the threshold of detection; this is one of the limitations of the available technology. These data do show that 95% of the heteroplasmic mutations detected in both tissues were non-pathogenic polymorphic variants, thus suggesting that pathogenic mtDNA mutations which occur in the germline or early development are selected against, and these non-pathogenic mtDNA mutations may make little contribution to the ageing phenotype. This demonstrates purifying selection in the human germline. Previous studies have shown this in mice by looking at transmission of mtDNA mutations through multiple generations [44], [45]; here we show purifying selection in humans by comparing the germline and somatic mtDNA mutations in different tissues from the same subjects. Recent evidence from the mouse has suggested that transmitted germline mtDNA mutations can cause premature ageing, perhaps by clonal expansion of these germline mtDNA mutations over time [46]. In our dataset 95% of the germline mtDNA mutations are benign and are unlikely to cause mitochondrial dysfunction and premature ageing, suggesting that there are differences in the dynamics of mtDNA transmission between these mutation prone mice and humans.

The somatic mtDNA mutations detected by NGS in the colonic epithelium only, are a combination of benign synonymous and polymorphic variants and non-synonymous potentially pathogenic variants, which we believe may begin to occur when mtDNA replication is re-initiated after the embryo has implanted into the uterine wall [33]. There was a significantly higher frequency of non-synonymous coding region somatic mtDNA mutations compared to the germline or early embryological mtDNA mutations which is evidence in support of the hypothesis that the somatic mutations occurred beyond any selective checkpoints, before expanding clonally to detectable levels.

The observations in this study are in agreement with evidence from epidemiological studies which suggest that damage arising early in human life can be an important modulator of outcomes in later life [47], [48]. Due to the time taken for clonal expansion of mtDNA mutations to occur in human cells, we hypothesise that late life *de novo* mtDNA mutational events make negligible contribution to the ageing phenotype and that early to mid-life mtDNA mutations are likely to be much more important.

## Materials and Methods

### Participants

Colorectal mucosal samples were collected from the same anatomical site (10 cm from the anal verge) from participants (n = 207, age range 17–78 years) undergoing colonoscopy for disturbed bowel function in whom no evidence of bowel disease was identified (BORICC 1 Study). Buccal epithelial scrapes were also collected concurrently from these subjects. The following subjects were also used in our previous work: BCC010, BCC011, BCC017, BCC022, BCC028, BCC085, BCC087, BCC088 [25].

### Ethics statement

Ethical approval was obtained from the Northumbria NHS Trust Local Research Ethics Committee (Project reference NLREC2/2001). All participants were fully informed and written consent obtained from them.

### Random Mutation Capture (RMC)

RMC was carried out essentially as previously described [25]. Briefly, mtDNA was extracted from colorectal mucosal biopsies and drop-dialysed using membrane filters (0.025 µm, Millipore) to extract any excess salts. One microlitre of mtDNA was digested with 100 U of *Taq*Iα (New England Biolabs) for 10 hours with the addition of 100 U every hour. MtDNA copy number was quantified by SYBR Green real-time PCR (Roche) targeting a template outside of a *Taq*Iα restriction site in *MTND5* (primers L12473–L12492 and H12573-H12554) Absolute quantification was carried out using the standard curve method. PCR was then carried out across a *Taq*Iα restriction site within the *MTCOI* gene (bp 6562–6565, primers L4636–L6455 and H6851–H6870). An average of 2500 copies of the target sequence (a total of 10000 target bases) was added to each PCR reaction. Following PCR each product was digested with 50 units of *Taq*Iα for 1 hour at 65°C, followed by 10 minutes at 80°C to inactivate the enzyme. Products were then subjected to electrophoresis through a 1.5% agarose gel for 1 hour at 200 V. All full length (488 bp) products were excised from the gel using a QIAquick Gel Extraction kit (Qiagen). These products were sequenced using ABI BigDye chemistries per standard manufacturer's protocols and analysed on an ABI3100 Genetic Analyser (Applied Biosystems). Sequences obtained were compared with the revised Cambridge Reference Sequence (GenBank accession number: NC_012920.1) using SeqScape software (Applied Biosystems). Mutation load was calculated by dividing the number of confirmed mutants by the total number of base pairs investigated.

### Validation of the RMC assay

To investigate the sensitivity and specificity of the RMC assay in our hands we generated a PCR product which contained a mtDNA mutation in the RMC site and one which was wild-type in the RMC site. A pCR-scriptTM Amp SK(+) cloning Kit (Stratagene) was used to clone the products following the manufacturer's protocol. Recombinant plasmids were identified by blue–white colour selection and the cultures grown up using a Qiaquick miniprep kit (Qiagen). The DNA was then extracted and quantified and the wild-type and mutant PCR products mixed at concentrations ranging from 100% wild-type to 100% mutant. The RMC assay was then carried out as above and the observed mutant fractions calculated and compared to the expected fractions (Table S4). There was no difference between observed and expected fractions, confirming the RMC assay to be both highly sensitive and specific.

### Next generation sequencing

Next generation sequencing (NGS) was carried out using an Ion Torrent Personal Genome Machine (Life Technologies, Paisley, UK) on whole mtDNA from the same colonic biopsies investigated by RMC and from buccal epithelia from the 8 youngest (<26 years) and 8 oldest (>70 years) subjects. To exclude the possibility of nuclear pseudogene amplification, extracted DNA was amplified in two overlapping 9 kb fragments using primers L2091–L2111 and H10649-H10629 (primer set 1), and L10085–L10104 and H2644-2625 (primer set 2), the specificity of which was established after observing no amplification from Rho Zero cells, cells depleted of their mtDNA by ethidium bromide treatment.

Long-range PCR amplicons were quantified on an Agilent 2100 Bioanalyzer with an Agilent DNA 12,000 kit (Agilent Technologies, Stockport, UK). Overlapping PCR fragments for each sample were combined in equimolar concentrations. Pooled amplicons (100 ng) were then fragmented, barcoded, size-selected and amplified using the IonXpress Plus Fragment Library kit, Ion Xpress Barcode Adapters and E-Gel SizeSelect 2% agarose gels (Life Technologies), according to the manufacturer's recommendations. Barcoded libraries were quantified with an Agilent Bioanalyzer DNA High Sensitivity kit then pooled (n = 16) in equimolar concentrations and diluted to 26 pM, prior to clonal amplification onto Ion Sphere Particles using the Ion OneTouch 1 System and the Ion OneTouch 200 Template kit v2 (Life Technologies), as per the manufacturer's instructions. Coated spheres were enriched on the Ion Torrent ES (Life Technologies) before loading onto Ion 318 sequencing chips (Life Technologies). Next-generation semiconductor sequencing was performed on an Ion Torrent Personal Genome Machine (Life Technologies). Fastq data files downloaded from the Torrent Server (version 3.6.2, Life Technologies) were analysed using NextGENe software (v2.3.0; SoftGenetics, State College, PA, USA).

### Quality control criteria for NGS

The background noise on the Ion Torrent PGM platform was quantified by extraction of DNA from a yeast clone which had been transfected with a plasmid (pRShmt) containing the entire human mtDNA [49] (kindly donated by Dr Brian Bigger (University of Manchester, UK)) which was then subjected to an identical PCR amplification and NGS protocol as the colonic biopsy and buccal scrape samples. On this basis any low level mtDNA variants detected in the cloned mtDNA are likely to be technical artefacts arising from the PCR and sequencing process and we could quantify the level of background noise and exclude this from the sample analysis. In addition the Ion Torrent platform may be prone to base calling errors in polynucleotide tracts, most often calling them as small insertions or deletions; therefore we restricted the analysis to base-pair substitutions only. Further quality control steps taken were; (1) only base substitutions with a quality score >20 were included in order to be confident that the calls were genuine, (2) observed variants had to be present in both forward and reverse reads at comparable frequencies with a 3-fold difference permitted to allow for the effects of a binomial sampling distribution at very low variant levels [28], (3) at least 3 reads were required for each variant, with a minimum total coverage of 600 reads per site. There was an unstable tract between base pairs 3902 and 3908 which repeatedly showed heteroplasmy levels between 1% and 5% in all of the samples and the plasmid control, as did a recognised variant at base pair 750. These mtDNA variants were deemed artefactual and removed from the analysis. Using these stringent criteria, there were no variants present at >0.65% in the cloned DNA template (Table S5). We took a conservative approach and only recorded mutations present at >0.8%. This approach ensured that any variants detected in the samples at levels of >0.8% are likely to be generated *in vivo* and be of biological origin. The published base-substitution error rate for mtDNA on the Ion Torrent PGM is 0.12% [50].

### Cyctochrome *c* oxidase/succinate dehydrogenase (COX/SDH) histochemistry

Colon samples were mounted for sectioning and frozen in isopentane previously cooled to −190°C in liquid nitrogen. Cryostat sections (12 µm) were cut onto glass slides and incubated in COX medium (100 µM cytochrome *c*, 4 mM diaminobenzidine tetrahydrochloride and 20 µg.ml^−1^ catalase in 0.2 M phosphate buffer pH 7.0) at 37°C for 50 minutes. Sections were washed in phosphate buffered saline, pH 7.4 (3×5 minutes) and incubated in SDH medium (130 mM sodium succinate, 200 µM phenazine methosulphate, 1 mM sodium azide, 1.5 mM nitroblue tetrazolium in 0.2 M phosphate buffer pH 7.0) at 37°C for 45 minutes. Finally, sections were washed in phosphate buffered saline, pH 7.4 (3×5 minutes), dehydrated in a graded ethanol series (70%, 95%, 2×100%), cleared in Histoclear (National Diagnostics, Atlanta, USA) and mounted in DPX.

## Supporting Information

Figure S1 RMC analysis with zero values removed.
(PDF)

Table S1 Random mutation capture raw data.
(PDF)

Table S2 Mitochondrial DNA (mtDNA) mutations detected by Ion Torrent Next Generation Sequencing in human colonic epithelium.
(PDF)

Table S3 Mitochondrial DNA (mtDNA) mutations detected by Ion Torrent Next Generation Sequencing in human buccal epithelium.
(PDF)

Table S4 Validation of the sensitivity and specificity of the RMC assay.
(PDF)

Table S5 MtDNA variants detected in the cloned mtDNA plasmid control.
(PDF)
