# Supplementary material for: Clonal Expansion of Early to Mid-Life Mitochondrial DNA Point Mutations Drives Mitochondrial Dysfunction during Human Ageing
Source: PLoS Genet. 2014 Sep 18;10(9):e1004620. doi: 10.1371/journal.pgen.1004620 (PMC4169240; doi:10.1371/journal.pgen.1004620)
Supplement: Figure S1 — RMC analysis with zero values removed. (PDF) [file pgen.1004620.s001.pdf]

Figure S1: RMC analysis with zero values removed.

A) Frequency of mtDNA mutations detected in human colorectal mucosa. Mutation frequency per base pair was measured by RMC in colorectal mucosal biopsies from 207 volunteers with no colorectal disease. When zero values were removed there was no correlation between mtDNA mutation frequency and age (Pearson correlation = 0.136 (p=0.07)). **B**) Frequency of mtDNA mutations detected in human colonic mucosa by RMC grouped by decade with zero values removed. There were no significant differences between any of the groups (p=0.46, One Way ANOVA).

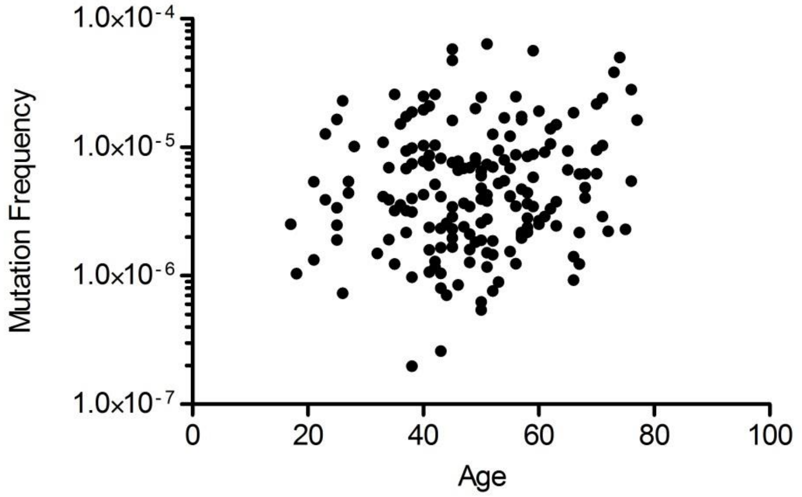

A

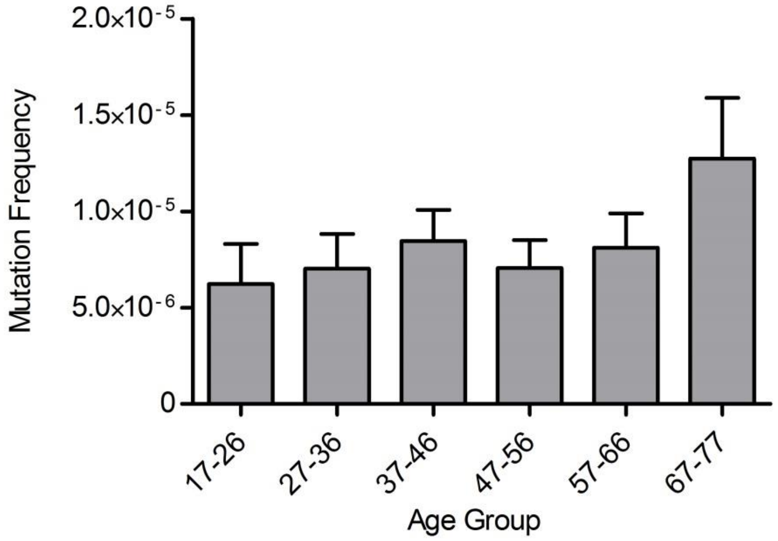

B
