# Supplementary material for: Clonal Expansion of Early to Mid-Life Mitochondrial DNA Point Mutations Drives Mitochondrial Dysfunction during Human Ageing
Source: PLoS Genet. 2014 Sep 18;10(9):e1004620. doi: 10.1371/journal.pgen.1004620 (PMC4169240; doi:10.1371/journal.pgen.1004620)
Supplement: Table S1 — Random mutation capture raw data. (PDF) [file pgen.1004620.s002.pdf]

Table S1: Random Mutation Capture raw data

Subject	Age	Copy number	Total Mutations	Mutation Frequency	6562 T>C	6562 T>A	6562 T>G	6563 C>A	6563 C>G	6563 C>T	6564 G>A	6564 G>C	6564 G>T	6565 A>C	6565 A>G	6565 A>T	Ins	Del
BCC001	29	241951	0	0	0	0	0	0	0	0	0	0	0	0	0	0	0	0
BCC004	70	419672	4	9.53125E-06	2	0	0	0	0	0	0	0	2	0	0	0	0	0
BCC005	30	565758	0	0	0	0	0	0	0	0	0	0	0	0	0	0	0	0
BCC007	35	216216	0	0	0	0	0	0	0	0	0	0	0	0	0	0	0	0
BCC008	62	902582	3	3.3238E-06	0	0	0	1	0	0	1	0	0	0	1	0	0	0
BCC010	46	1175608	1	8.50624E-07	0	0	0	0	0	0	1	0	0	0	0	0	0	0
BCC011	52	1316285	1	7.59714E-07	0	0	0	0	0	0	0	0	0	0	1	0	0	0
BCC012	68	585337	0	0	0	0	0	0	0	0	0	0	0	0	0	0	0	0
BCC013	55	647954	1	1.54332E-06	0	0	0	0	0	0	1	0	0	0	0	0	0	0
BCC014	40	120296	3	2.49385E-05	0	0	0	0	0	0	3	0	0	0	0	0	0	0
BCC015	42	116424	3	2.57679E-05	0	0	0	0	0	1	0	0	2	0	0	0	0	0
BCC017	45	1360432	0	0	0	0	0	0	0	0	0	0	0	0	0	0	0	0
BCC019	39	305096	0	0	0	0	0	0	0	0	0	0	0	0	0	0	0	0
BCC021	74	340768	17	4.98873E-05	0	0	0	0	0	0	17	0	0	0	0	0	0	0
BCC022	60	794205	2	2.51824E-06	0	0	0	1	0	0	0	0	0	0	0	0	0	1
BCC023	66	708747	1	1.41094E-06	0	0	0	1	0	0	0	0	0	0	0	0	0	0
BCC024	50	3048056	12	3.93694E-06	8	0	0	0	0	0	0	0	0	0	3	1	0	0
BCC025	63	1226169	3	2.44664E-06	0	0	0	0	0	0	2	0	1	0	0	0	0	0
BCC027	49	349448	7	2.00316E-05	0	0	0	0	0	3	2	0	1	0	1	0	0	0
BCC028	48	732463	0	0	0	0	0	0	0	0	0	0	0	0	0	0	0	0
BCC029	68	496584	2	4.02752E-06	0	0	0	0	0	1	0	0	0	0	1	0	0	0
BCC030	65	600072	4	6.66587E-06	0	0	0	0	0	1	1	0	0	0	2	0	0	0
BCC031	42	778800	4	5.13611E-06	0	0	0	0	0	0	3	0	1	0	0	0	0	0
BCC032	56	161832	4	2.4717E-05	2	0	0	0	0	0	1	0	0	0	1	0	0	0
BCC033	55	238832	1	4.18704E-06	0	0	0	0	0	0	0	0	1	0	0	0	0	0
BCC044	72	450523	1	2.21964E-06	0	0	0	0	0	0	0	0	0	0	1	0	0	0
BCC046	52	1139160	8	7.02272E-06	0	0	0	0	0	0	5	0	1	0	2	0	0	0
BCC047	76	249304	7	2.80782E-05	1	0	0	0	0	0	2	0	4	0	0	0	0	0
BCC048	60	209176	4	1.91227E-05	0	0	0	0	0	0	3	0	0	0	0	0	0	1
BCC049	45	1041392	3	2.88076E-06	0	0	0	0	0	0	1	0	1	0	1	0	0	0
BCC055	61	765160	7	9.14841E-06	0	0	0	0	0	1	5	0	0	0	1	0	0	0
BCC056	49	914732	7	7.65251E-06	1	0	0	0	0	1	5	0	0	0	0	0	0	0
BCC057	47	436656	3	6.8704E-06	0	0	0	0	0	0	2	0	1	0	0	0	0	0
BCC058	43	556160	0	0	0	0	0	0	0	0	0	0	0	0	0	0	0	0
BCC059	47	484440	0	0	0	0	0	0	0	0	0	0	0	0	0	0	0	0
BCC060	72	161119	0	0	0	0	0	0	0	0	0	0	0	0	0	0	0	0
BCC061	43	428912	1	2.33148E-06	0	0	0	0	0	0	0	0	0	0	1	0	0	0
BCC062	48	788040	1	1.26897E-06	0	0	0	0	0	0	0	0	0	0	1	0	0	0
BCC063	42	2572064	3	1.16638E-06	0	0	0	0	0	1	2	0	0	0	0	0	0	0
BCC064	38	1023352	1	9.77181E-07	0	0	0	0	0	0	1	0	0	0	0	0	0	0
BCC065	43	1245552	1	8.02857E-07	1	0	0	0	0	0	0	0	0	0	0	0	0	0
BCC066	32	2181960	0	0	0	0	0	0	0	0	0	0	0	0	0	0	0	0
BCC067	57	461120	1	2.16863E-06	0	0	0	0	0	0	0	0	0	0	1	0	0	0
BCC068	18	1926760	2	1.03801E-06	0	0	0	0	0	0	1	0	0	0	1	0	0	0
BCC069	66	332200	0	0	0	0	0	0	0	0	0	0	0	0	0	0	0	0
BCC070	42	775104	1	1.29015E-06	0	0	0	0	0	0	0	0	0	0	1	0	0	0
BCC071	66	2161280	2	9.25378E-07	1	0	0	0	0	0	0	0	0	0	1	0	0	0
BCC072	67	1618936	2	1.23538E-06	1	0	0	0	0	0	1	0	0	0	0	0	0	0

Subject	Age	Copy number	Total Mutations	Mutation Frequency	6562 T>C	6562 T>A	6562 T>G	6563 C>A	6563 C>G	6563 C>T	6564 G>A	6564 G>C	6564 G>T	6565 A>C	6565 A>G	6565 A>T	Ins	Del
BCC073	38	1437216	0	0	0	0	0	0	0	0	0	0	0	0	0	0	0	0
BCC075	52	2147640	4	1.86251E-06	0	0	0	0	0	0	1	0	0	0	3	0	0	0
BCC076	58	419848	1	2.38181E-06	0	0	0	0	0	0	0	0	0	0	0	0	0	1
BCC077	40	1029096	8	7.77381E-06	0	0	0	0	0	1	5	1	0	0	1	0	0	0
BCC078	60	745160	2	2.68399E-06	1	0	0	0	0	0	1	0	0	0	0	0	0	0
BCC079	40	1067019	11	1.03091E-05	7	0	0	0	0	1	0	0	1	0	2	0	0	0
BCC080	45	4787782	8	1.67092E-06	1	0	0	0	0	0	4	0	0	0	3	0	0	0
BCC081	36	131572	2	1.52008E-05	0	0	0	0	0	0	1	0	0	0	1	0	0	0
BCC082	59	550669	0	0	0	0	0	0	0	0	0	0	0	0	0	0	0	0
BCC083	45	433288	1	2.30793E-06	0	0	0	0	0	0	0	0	0	0	0	0	0	1
BCC084	52	1046280	0	0	0	0	0	0	0	0	0	0	0	0	0	0	0	0
BCC085	51	5135368	6	1.16837E-06	2	0	0	1	0	0	2	0	1	0	0	0	0	0
BCC086	63	629702	0	0	0	0	0	0	0	0	0	0	0	0	0	0	0	0
BCC087	64	703448	0	0	0	0	0	0	0	0	0	0	0	0	0	0	0	0
BCC088	57	958437	2	2.08673E-06	0	0	0	0	0	1	0	0	1	0	0	0	0	0
BCC096	66	431637	8	1.85341E-05	4	0	0	1	0	0	3	0	0	0	0	0	0	0
BCC097	41	420200	1	2.37982E-06	0	0	0	0	0	0	1	0	0	0	0	0	0	0
BCC098	17	396000	1	2.52525E-06	0	0	0	0	0	0	1	0	0	0	0	0	0	0
BCC099	59	338888	3	8.85248E-06	1	0	0	0	0	0	0	0	2	0	0	0	0	0
BCC100	52	317504	4	1.25983E-05	1	0	0	0	0	0	2	0	1	0	0	0	0	0
BCC101	62	574816	8	1.39175E-05	2	0	0	0	0	0	3	0	1	0	2	0	0	0
BCC102	59	106304	6	5.64419E-05	1	0	0	0	0	0	0	0	5	0	0	0	0	0
BCC103	51	204512	13	6.3566E-05	0	0	0	0	0	0	10	0	1	0	1	1	0	0
BCC104	42	577544	6	1.03888E-05	0	0	0	1	0	1	2	0	2	0	0	0	0	0
BCC106	27	1109152	6	5.40954E-06	1	0	0	0	0	0	5	0	0	0	0	0	0	0
BCC107	55	736553	9	1.22191E-05	0	0	0	0	0	1	0	0	0	0	8	0	0	0
BCC108	58	2140160	6	2.80353E-06	1	0	0	0	0	0	1	0	3	0	1	0	0	0
BCC109	73	208120	8	3.84394E-05	0	0	0	0	0	0	8	0	0	0	0	0	0	0
BCC110	24	174240	4	2.29568E-05	1	0	0	1	0	0	1	1	0	0	0	0	0	0
BCC111	63	1063216	4	3.76217E-06	0	0	0	1	0	0	3	0	0	0	0	0	0	0
BCC112	25	1580040	3	1.89869E-06	0	0	0	0	0	0	2	0	0	0	1	0	0	0
BCC113	78	122879	2	1.62762E-05	0	0	0	0	0	0	2	0	0	0	0	0	0	0
BCC114	58	1373064	3	2.18489E-06	0	0	0	0	0	0	2	0	0	0	0	0	0	1
BCC115	51	680152	5	7.3513E-06	0	0	0	0	0	0	1	0	0	0	0	0	0	4
BCC116	41	334664	7	2.09165E-05	0	0	0	1	0	0	5	0	0	0	1	0	0	0
BCC117	25	486384	8	1.64479E-05	2	0	0	0	0	0	1	0	0	0	5	0	0	0
BCC118	63	466830	7	1.49947E-05	1	0	0	0	0	0	2	0	1	0	3	0	0	0
BCC119	68	482405	3	6.21884E-06	0	0	0	1	0	1	0	0	0	0	1	0	0	0
BCC120	32	671375	1	1.48948E-06	0	0	0	0	0	0	1	0	0	0	0	0	0	0
BCC121	40	699938	0	0	0	0	0	0	0	0	0	0	0	0	0	0	0	0
BCC122	37	535420	5	9.33846E-06	0	0	0	0	0	0	2	0	1	0	1	0	0	1
BCC123	50	449020	11	2.44978E-05	0	0	0	0	0	0	6	0	2	0	0	0	0	3
BCC124	51	661545	1	1.51161E-06	0	0	0	0	0	0	0	0	1	0	0	0	0	0
BCC125	49	606984	5	8.23745E-06	0	0	0	1	0	1	3	0	0	0	0	0	0	0
BCC126	43	603530	1	1.65692E-06	0	0	0	0	0	0	0	0	0	0	0	0	1	0
BCC127	47	415773	1	2.40516E-06	0	0	0	0	0	0	1	0	0	0	0	0	0	0
BCC128	59	869051	3	3.45204E-06	0	0	0	0	0	1	1	0	0	0	0	0	0	1
BCC129	49	548732	1	1.82238E-06	0	0	0	0	0	1	0	0	0	0	0	0	0	0
BCC130	58	676326	3	4.43573E-06	0	0	0	0	0	0	3	0	0	0	0	0	0	0
BCC131	58	731821	0	0	0	0	0	0	0	0	0	0	0	0	0	0	0	0
BCC132	57	507194	1	1.97163E-06	0	0	0	0	0	0	1	0	0	0	0	0	0	0

Subject	Age	Copy number	Total Mutations	Mutation Frequency	6562 T>C	6562 T>A	6562 T>G	6563 C>A	6563 C>G	6563 C>T	6564 G>A	6564 G>C	6564 G>T	6565 A>C	6565 A>G	6565 A>T	Ins	Del
BCC133	55	483138	2	4.13961E-06	0	0	0	0	0	0	1	0	1	0	0	0	0	0
BCC134	63	470642	0	0	0	0	0	0	0	0	0	0	0	0	0	0	0	0
BCC135	38	318497	1	3.13974E-06	0	0	0	0	0	0	0	0	0	0	1	0	0	0
BCC136	44	780589	2	2.56217E-06	0	0	0	0	0	2	0	0	0	0	0	0	0	0
BCC137	50	623936	3	4.80819E-06	0	0	0	0	0	1	1	0	0	0	1	0	0	0
BCC138	59	606932	0	0	0	0	0	0	0	0	0	0	0	0	0	0	0	0
BCC139	71	331806	8	2.41105E-05	1	0	0	0	0	0	4	0	3	0	0	0	0	0
BCC140	72	156816	0	0	0	0	0	0	0	0	0	0	0	0	0	0	0	0
BCC141	57	2485912	5	2.01133E-06	3	0	0	0	0	0	1	0	0	0	1	0	0	0
BCC142	61	1014728	0	0	0	0	0	0	0	0	0	0	0	0	0	0	0	0
BCC144	76	551056	3	5.44409E-06	0	0	0	0	0	0	2	0	0	0	0	1	0	0
BCC145	55	145464	1	6.87455E-06	0	0	0	0	0	0	1	0	0	0	0	0	0	0
BCC146	43	3846304	1	2.5999E-07	0	0	0	0	0	0	1	0	0	0	0	0	0	0
BCC147	21	1500752	2	1.33267E-06	0	0	0	1	0	0	0	0	0	0	1	0	0	0
BCC148	48	1254440	2	1.59434E-06	2	0	0	0	0	0	0	0	0	0	0	0	0	0
BCC149	37	1383272	3	2.16877E-06	0	0	0	0	0	0	3	0	0	0	0	0	0	0
BCC150	37	671176	0	0	0	0	0	0	0	0	0	0	0	0	0	0	0	0
BCC151	56	806784	1	1.23949E-06	0	0	0	0	0	1	0	0	0	0	0	0	0	0
BCC152	45	1018072	2	1.9645E-06	0	0	0	0	0	0	1	0	1	0	0	0	0	0
BCC153	46	1671648	13	7.77676E-06	0	0	0	0	0	6	7	0	0	0	0	0	0	0
BCC154	50	3693008	2	5.41564E-07	0	0	0	0	0	0	0	0	1	0	1	0	0	0
BCC155	26	1370512	1	7.29654E-07	0	0	0	0	0	0	1	0	0	0	0	0	0	0
BCC156	37	150832	0	0	0	0	0	0	0	0	0	0	0	0	0	0	0	0
BCC157	61	345312	1	2.89593E-06	0	0	0	0	0	0	1	0	0	0	0	0	0	0
BCC158	34	522192	1	1.915E-06	0	0	0	0	0	0	1	0	0	0	0	0	0	0
BCC160	68	410872	2	4.8677E-06	0	0	0	0	0	1	1	0	0	0	0	0	0	0
BCC161	37	935792	3	3.20584E-06	1	0	0	1	0	0	0	0	0	1	0	0	0	0
BCC162	35	935968	3	3.20524E-06	0	0	0	0	0	0	2	0	0	0	1	0	0	0
BCC163	27	1587960	7	4.40817E-06	0	0	0	0	0	0	6	0	0	0	1	0	0	0
BCC164	43	1955712	16	8.18116E-06	0	0	0	0	0	0	16	0	0	0	0	0	0	0
BCC165	25	2417624	6	2.48178E-06	1	0	0	0	0	0	3	0	0	0	2	0	0	0
BCC166	50	1598432	1	6.25613E-07	0	0	0	0	0	0	0	0	0	0	1	0	0	0
BCC167	46	752752	5	6.64229E-06	0	0	0	1	0	1	0	0	1	0	2	0	0	0
BCC168	66	888624	0	0	0	0	0	0	0	0	0	0	0	0	0	0	0	0
BCC169	50	1333288	8	6.0002E-06	1	0	0	0	0	0	4	0	1	0	2	0	0	0
BCC171	44	1416184	1	7.06123E-07	0	0	0	0	0	0	1	0	0	0	0	0	0	0
BCC172	48	721424	5	6.93074E-06	4	0	0	0	0	1	0	0	0	0	0	0	0	0
BCC173	41	4407920	7	1.58805E-06	0	0	0	0	0	1	3	0	0	0	2	0	0	1
BCC174	51	3048320	13	4.26464E-06	0	0	0	0	0	2	9	0	0	0	2	0	0	0
BCC175	51	723639	2	2.76381E-06	0	0	0	0	0	0	1	0	0	0	0	1	0	0
BCC176	67	1778480	11	6.18506E-06	1	0	0	0	0	4	5	0	0	0	1	0	0	0
BCC177	50	2458368	16	6.50838E-06	0	0	0	0	0	0	15	0	0	0	1	0	0	0
BCC178	43	959464	1	1.04225E-06	0	0	0	0	0	0	0	0	1	0	0	0	0	0
BCC179	40	664928	13	1.9551E-05	1	0	0	0	0	0	12	0	0	0	0	0	0	0
BCC180	25	1181400	4	3.38581E-06	0	0	0	0	0	0	4	0	0	0	0	0	0	0
BCC181	57	305101	5	1.6388E-05	5	0	0	0	0	0	0	0	0	0	0	0	0	0
BCC182	70	802317	5	6.23195E-06	0	0	0	0	0	0	4	0	1	0	0	0	0	0
BCC183	50	1057011	2	1.89213E-06	0	0	0	0	0	0	1	0	0	0	1	0	0	0
BCC184	52	2054773	3	1.46002E-06	0	0	0	0	0	1	1	0	0	0	1	0	0	0
BCC185	56	575019	2	3.47814E-06	0	0	0	0	0	0	1	0	0	0	1	0	0	0
BCC187	34	1793281	7	3.90346E-06	0	0	0	0	0	0	5	0	0	0	2	0	0	0

Subject	Age	Copy number	Total Mutations	Mutation Frequency	6562 T>C	6562 T>A	6562 T>G	6563 C>A	6563 C>G	6563 C>T	6564 G>A	6564 G>C	6564 G>T	6565 A>C	6565 A>G	6565 A>T	Ins	Del
BCC188	51	2094218	8	3.82004E-06	0	0	0	0	0	2	5	0	0	0	1	0	0	0
BCC189	59	171116	1	5.844E-06	0	0	0	0	0	0	0	0	1	0	0	0	0	0
BCC190	38	912541	9	9.86257E-06	0	0	0	0	0	0	9	0	0	0	0	0	0	0
BCC191	56	1031459	9	8.7255E-06	0	0	0	0	0	7	1	1	0	0	0	0	0	0
BCC192	53	1117337	1	8.94985E-07	0	0	0	0	0	0	1	0	0	0	0	0	0	0
BCC193	45	63064	3	4.75707E-05	0	0	0	0	0	0	3	0	0	0	0	0	0	0
BCC194	33	241280	1	4.14456E-06	0	0	0	0	0	0	0	0	0	1	0	0	0	0
BCC195	38	1208480	9	7.44737E-06	0	0	0	0	0	1	8	0	0	0	0	0	0	0
BCC196	43	241940	1	4.13325E-06	0	0	0	0	0	0	1	0	0	0	0	0	0	0
BCC197	53	3250274	17	5.23033E-06	0	0	0	0	0	0	0	0	0	0	17	0	0	0
BCC198	38	1000012	4	3.99995E-06	0	0	0	1	0	0	2	0	1	0	0	0	0	0
BCC199	65	535191	5	9.34246E-06	1	0	0	0	0	0	1	0	3	0	0	0	0	0
BCC200	41	580046	5	8.62E-06	1	0	0	0	0	0	3	0	0	0	0	0	0	1
BCC201	38	5066210	1	1.97386E-07	0	0	0	0	0	0	1	0	0	0	0	0	0	0
BCC202	50	1554201	4	2.57367E-06	0	0	0	0	0	1	1	0	1	0	1	0	0	0
BCC203	70	646234	14	2.1664E-05	1	0	0	0	0	0	13	0	0	0	0	0	0	0
BCC204	57	633001	11	1.73775E-05	8	0	0	0	0	0	2	0	0	0	1	0	0	0
BCC205	23	157090	2	1.27316E-05	0	0	0	0	0	0	1	0	1	0	0	0	0	0
BCC206	54	354894	6	1.69065E-05	0	0	0	1	0	0	0	0	0	5	0	0	0	0
BCC207	62	563880	6	1.06406E-05	4	0	0	0	0	0	0	0	0	0	2	0	0	0
BCC208	54	125396	1	7.97472E-06	0	0	0	0	0	0	1	0	0	0	0	0	0	0
BCC209	40	963051	0	0	0	0	0	0	0	0	0	0	0	0	0	0	0	0
BCC210	37	440776	3	6.80618E-06	0	0	0	0	0	0	3	0	0	0	0	0	0	0
BCC211	35	4060225	5	1.23146E-06	0	0	0	2	0	0	0	0	3	0	0	0	0	0
BCC212	45	263020	2	7.60398E-06	0	0	0	1	0	0	1	0	0	0	0	0	0	0
BCC213	71	692250	2	2.88913E-06	0	0	0	0	0	0	1	0	0	0	1	0	0	0
BCC214	48	947081	2	2.11175E-06	1	0	0	0	0	0	0	0	0	0	1	0	0	0
BCC215	40	466850	2	4.28403E-06	1	0	0	0	0	0	1	0	0	0	0	0	0	0
BCC216	48	1449838	5	3.44866E-06	0	0	0	0	0	1	4	0	0	0	0	0	0	0
BCC217	36	1690522	6	3.5492E-06	0	0	0	0	0	0	3	0	0	1	0	0	0	2
BCC218	36	983496	0	0	0	0	0	0	0	0	0	0	0	0	0	0	0	0
BCC219	42	718803	0	0	0	0	0	0	0	0	0	0	0	0	0	0	0	0
BCC220	33	91281	1	1.09552E-05	0	0	0	0	0	0	0	0	0	0	0	0	0	1
BCC221	47	546922	2	3.65683E-06	0	0	0	1	0	0	1	0	0	0	0	0	0	0
BCC222	75	1738176	4	2.30126E-06	0	0	0	1	0	0	2	0	0	0	0	0	0	1
BCC223	54	365755	2	5.46815E-06	0	0	0	0	0	0	0	0	0	0	2	0	0	0
BCC225	28	590137	6	1.01671E-05	1	0	0	0	0	0	3	0	0	0	2	0	0	0
BCC226	57	849514	4	4.70858E-06	0	0	0	0	0	0	3	0	0	0	1	0	0	0
BCC227	67	921423	2	2.17056E-06	0	0	0	0	0	0	1	0	0	0	1	0	0	0
BCC228	60	1118317	0	0	0	0	0	0	0	0	0	0	0	0	0	0	0	0
BCC229	34	143862	1	6.9511E-06	0	0	0	0	0	1	0	0	0	0	0	0	0	0
BCC230	58	548066	2	3.6492E-06	0	0	0	2	0	0	0	0	0	0	0	0	0	0
BCC232	45	870232	3	3.44736E-06	1	0	0	0	0	0	1	0	1	0	0	0	0	0
BCC233	37	57733	1	1.73213E-05	0	0	0	0	0	1	0	0	0	0	0	0	0	0
BCC242	35	388049	10	2.577E-05	0	0	0	0	0	0	1	0	9	0	0	0	0	0
BCC243	38	106755	2	1.87344E-05	0	0	0	0	0	0	2	0	0	0	0	0	0	0
BCC244	23	256688	1	3.89578E-06	0	0	0	0	0	1	0	0	0	0	0	0	0	0
BCC245	21	558114	3	5.37524E-06	1	0	0	0	0	0	2	0	0	0	0	0	0	0
BCC247	45	172101	10	5.81053E-05	0	0	0	0	0	0	10	0	0	0	0	0	0	0
BCC248	58	2355544	20	8.49061E-06	0	0	0	0	0	0	20	0	0	0	0	0	0	0
BCC249	41	1386692	10	7.21141E-06	0	0	0	0	0	0	10	0	0	0	0	0	0	0

Subject	Age	Copy number	Total Mutations	Mutation Frequency	6562 T>C	6562 T>A	6562 T>G	6563 C>A	6563 C>G	6563 C>T	6564 G>A	6564 G>C	6564 G>T	6565 A>C	6565 A>G	6565 A>T	Ins	Del
BCC250	45	618249	10	1.61747E-05	0	0	0	0	0	0	10	0	0	0	0	0	0	0
BCC251	41	936018	1	1.06836E-06	0	0	0	0	0	0	1	0	0	0	0	0	0	0
BCC252	53	527969	5	9.47025E-06	0	0	0	0	0	0	5	0	0	0	0	0	0	0
BCC260	71	290770	3	1.03174E-05	0	0	0	0	0	0	3	0	0	0	0	0	0	0
BCC265	72	631719	0	0	0	0	0	0	0	0	0	0	0	0	0	0	0	0
BCC266	74	611718	22	3.59643E-05	0	0	0	0	0	0	22	0	0	0	0	0	0	0
