# Supplementary material for: Clonal Expansion of Early to Mid-Life Mitochondrial DNA Point Mutations Drives Mitochondrial Dysfunction during Human Ageing
Source: PLoS Genet. 2014 Sep 18;10(9):e1004620. doi: 10.1371/journal.pgen.1004620 (PMC4169240; doi:10.1371/journal.pgen.1004620)
Supplement: Table S2 — Mitochondrial DNA (mtDNA) mutations detected by Ion Torrent Next Generation Sequencing in human colonic epithelium. (PDF) [file pgen.1004620.s003.pdf]

Table S2: Mitochondrial DNA (mtDNA) mutations detected by Ion Torrent Next Generation Sequencing in human colonic epithelium
MtDNA mutations highlighted in red are present in both buccal and colonic epithelium in that subject.
(Germline Ref) is the reference base for that individual subject which is different from the rCRS
rCRS: Revised Cambridge Reference Sequence

Participant ID	Age	Base Position	Reference Base (rCRS)	Mutant Base	Mutant Frequency	Heteroplasmy	Gene	Amino Acid Change	Reported Polymorphism
BCC044	72	150	C	T	34	2.08	Non-coding	~	Yes
BCC044	72	204	T	C	38	2.55	Non-coding	~	Yes
BCC044	72	215	G (Germline ref)	A	11	0.85	Non-coding	~	Yes
BCC044	72	1255	T	C	20	0.93	MT-RNR1	~	Yes
BCC044	72	1777	A	G	8	0.93	MT-RNR2	~	No
BCC044	72	2148	A	G	11	0.82	MT-RNR2	~	No
BCC044	72	2315	A	G	11	0.8	MT-RNR2	~	Yes
BCC044	72	4592	T	G	8	0.98	MT-ND2	Synonymous	No
BCC044	72	7028	T (Germline Ref)	C	10	0.87	MT-CO1	Synonymous	Yes
BCC044	72	7372	T	C	6	1.01	MT-CO1	p.M490T	Yes
BCC044	72	12540	A	G	8	1.02	MT-ND5	Synonymous	Yes
BCC047	76	93	A	G	77	1.58	Non-coding	~	Yes
BCC047	76	1598	G	A	328	7.51	MT-RNR1	~	Yes
BCC047	76	1985	G	A	63	0.95	MT-RNR2	~	No
BCC047	76	2143	G	A	37	0.8	MT-RNR2	~	No
BCC047	76	2333	G	A	51	1.01	MT-RNR2	~	No
BCC047	76	2733	G	A	54	0.87	MT-RNR2	~	No
BCC047	76	3091	G	A	421	6.73	MT-RNR2	~	No
BCC047	76	12722	C (Germline Ref)	T	87	1.27	MT-ND5	p.P129K	No
BCC047	76	13480	G	A	25	0.92	MT-ND5	Synonymous	No
BCC047	76	13540	T	C	57	2.65	MT-ND5	p.S402P	No
BCC047	76	14200	T	C	8	1.03	MT-ND6	Synonymous	Yes
BCC047	76	14985	G	A	589	10.11	MT-CYB	p.A80H	No
BCC047	76	15093	G	A	910	10.92	MT-CYB	p.G116D	No
BCC047	76	16034	G	A	70	0.9	Non-coding	~	No
BCC098	17	1598	G	A	37	0.86	MT-RNR1	~	Yes
BCC098	17	8518	A	G	5	0.81	MT-ATP8	Synonymous	Yes
BCC098	17	12308	G (Germline ref)	A	36	1.4	MT-TL2	~	Yes
BCC098	17	14750	A	G	4	0.81	MT-CYB	p.T2A	Yes
BCC110	24	3915	A (Germline ref)	G	39	1.08	MT-ND1	Synonymous	Yes
BCC110	24	11253	C (Germline Ref)	T	14	1.09	MT-ND4	Synonymous	Yes
BCC110	24	12746	T	C	85	2.04	MT-ND5	p.K137P	No
BCC110	24	16362	C (Germline Ref)	T	14	0.88	Non-coding	~	Yes
BCC112	25	4769	G (Germline ref)	A	28	1	MT-ND2	Synonymous	Yes
BCC112	25	5782	T	C	9	0.89	MT-TC	~	Yes
BCC112	25	8994	A (Germline ref)	G	4	1.17	MT-ATP6	Synonymous	Yes
BCC112	25	13917	A	G	7	0.92	MT-ND5	Synonymous	Yes
BCC112	25	14053	G (Germline ref)	A	9	2.63	MT-ND5	Synonymous	Yes
BCC113	78	185	A (Germline ref)	G	206	5.58	Non-coding	~	Yes
BCC113	78	189	A	G	43	1.17	Non-coding	~	Yes

Participant ID	Age	Base Position	Reference Base (rCRS)	Mutant Base	Mutant Frequency	Heteroplasmy	Gene	Amino Acid Change	Reported Polymorphism
BCC113	78	1731	A	G	58	4.65	MT-RNR2	~	No
BCC113	78	1966	G	A	82	2	MT-RNR2	~	No
BCC113	78	2778	T	C	23	0.91	MT-RNR2	~	Yes
BCC113	78	3880	G	A	11	0.86	MT-ND1	p.E192K	No
BCC113	78	9024	A	G	6	0.85	MT-ATP6	Synonymous	Yes
BCC113	78	10325	G	A	39	1.33	MT-ND3	Synonymous	Yes
BCC113	78	11456	G	A	25	0.88	MT-ND4	p.A233T	No
BCC113	78	12287	T	G	87	2.81	MT-TL2	~	No
BCC113	78	12519	T	C	30	1.85	MT-ND5	Synonymous	Yes
BCC113	78	15452	A (Germline ref)	C	6	0.99	MT-CYB	p.I236K	No
BCC113	78	15757	G (Germline ref)	A	36	1.28	MT-CYB	Synonymous	Yes
BCC113	78	16034	G	A	37	0.8	Non-coding	~	No
BCC113	78	16093	C (Germline Ref)	T	424	12.88	Non-coding	~	Yes
BCC113	78	16355	C	T	73	3.85	Non-coding	~	Yes
BCC117	25	7363	A	G	6	0.99	MT-CO1	p.E487G	Yes
BCC117	25	12546	A	G	17	0.85	MT-ND5	Synonymous	No
BCC117	25	12584	A	G	21	0.8	MT-ND5	p.D83G	No
BCC117	25	14202	A	G	4	0.8	MT-ND6	p.W158R	No
BCC165	25	10992	C (Germline Ref)	T	4	0.83	MT-ND4	p.T78M	No
BCC165	25	12308	G (Germline ref)	A	18	1.35	MT-TL2	~	Yes
BCC165	25	15140	G	C	33	1.1	MT-CYB	p.V132L	No
BCC180	25	5059	A	G	8	0.8	MT-ND2	p.N197S	No
BCC205	23	3352	G	A	5	1.11	MT-ND1	p.A16T	No
BCC205	23	5874	T	C	7	0.95	MT-TY	~	No
BCC205	23	5973	G	A	20	2.6	MT-CO1	p.A24T	Yes
BCC205	23	7058	T	C	6	0.84	MT-CO1	Synonymous	Yes
BCC205	23	7295	A	G	6	0.8	MT-CO1	Synonymous	Yes
BCC205	23	7961	T	C	5	0.88	MT-CO2	Synonymous	Yes
BCC205	23	9926	A	G	8	0.83	MT-CO3	Synonymous	Yes
BCC213	71	653	G	A	38	2.05	MT-RNR1	~	No
BCC213	71	3424	G	A	27	1.75	MT-ND1	p.V40M	No
BCC213	71	11921	T	C	14	0.93	MT-ND4	p.W388R	No
BCC213	71	15713	T	C	26	1.12	MT-CYB	Synonymous	Yes
BCC213	71	15928	G	A	79	1.95	MT-TT	~	Yes
BCC222	75	146	T	C	56	2.17	Non-coding	~	Yes
BCC222	75	204	T	C	23	1	Non-coding	~	Yes
BCC222	75	3593	T	C	4	0.97	MT-ND1	p.V96A	Yes
BCC222	75	4510	T	C	37	1.5	MT-ND2	p.F14S	No
BCC222	75	10685	G	A	10	0.83	MT-ND4L	Synonymous	Yes
BCC222	75	12345	G	A	7	0.9	MT-ND5	Synonymous	No
BCC222	75	12701	T	C	31	1.08	MT-ND5	p.K122P	No
BCC222	75	15234	G	A	51	1.71	MT-CYB	Synonymous	No
BCC222	75	16023	G	A	46	1.22	Non-coding	~	No
BCC244	23	11251	G (Germline ref)	A	15	0.81	MT-ND4	Synonymous	Yes
BCC244	23	12007	A (Germline ref)	G	32	1.84	MT-ND4	Synonymous	Yes

Participant ID	Age	Base Position	Reference Base (rCRS)	Mutant Base	Mutant Frequency	Heteroplasmy	Gene	Amino Acid Change	Reported Polymorphism
BCC244	23	14692	A	G	7	1	MT-TE	~	Yes
BCC260	71	2342	T	C	51	1.41	MT-RNR2	~	No
BCC260	71	2903	T	C	12	1.35	MT-RNR2	~	Yes
BCC260	71	4721	A	G	5	1.19	MT-ND2	Synonymous	Yes
BCC260	71	5981	T	C	6	1.01	MT-CO1	Synonymous	Yes
BCC260	71	6141	A	G	7	0.85	MT-CO1	p.N80D	No
BCC260	71	6216	T	C	6	0.83	MT-CO1	Synonymous	Yes
BCC260	71	7028	T (Germline Ref)	C	6	0.84	MT-CO1	Synonymous	Yes
BCC260	71	8152	A (Germline ref)	G	6	0.9	MT-CO2	Synonymous	Yes
BCC260	71	9789	T	C	5	0.89	MT-CO3	p.S195P	No
BCC260	71	10569	G	T	6	0.91	MT-ND4L	Synonymous	No
BCC260	71	15140	G	A	86	1.05	MT-CYB	p.V132L	No
BCC265	72	189	A	G	21	0.83	Non-coding	~	Yes
BCC265	72	11334	A	G	19	1.07	MT-ND4	p.N192S	No
BCC265	72	13198	G	A	16	0.91	MT-ND5	p.A288T	Yes
BCC265	72	14865	G	A	29	2.63	MT-CYB	p.C40T	Yes
BCC266	74	2553	G	A	64	1.18	MT-RNR2	~	No
BCC266	74	9714	G	A	56	2.54	MT-CO3	p.G170S	No
BCC266	74	10586	G	A	26	0.96	MT-ND4L	Synonymous	Yes
BCC266	74	11456	G	A	20	1.04	MT-ND4	p.A233T	No
BCC266	74	11834	T	C	27	1.24	MT-ND4	p.W359R	No
BCC266	74	13818	T	C	7	0.86	MT-ND5	Synonymous	Yes
BCC266	74	16027	T	C	58	1.51	Non-coding	~	No
BCC266	74	16035	G	A	88	2.35	Non-coding	~	No
