# Supplementary material for: Clonal Expansion of Early to Mid-Life Mitochondrial DNA Point Mutations Drives Mitochondrial Dysfunction during Human Ageing
Source: PLoS Genet. 2014 Sep 18;10(9):e1004620. doi: 10.1371/journal.pgen.1004620 (PMC4169240; doi:10.1371/journal.pgen.1004620)
Supplement: Table S3 — Mitochondrial DNA (mtDNA) mutations detected by Ion Torrent Next Generation Sequencing in human buccal epithelium. (PDF) [file pgen.1004620.s004.pdf]

Table S3: Mitochondrial DNA (mtDNA) mutations detected by Ion Torrent Next Generation Sequencing in human buccal epithelium

MtDNA mutations highlighted in red are present in both buccal and colonic epitheluim in that subject.

(Germline Ref) is the reference base for that individual subject which is different from the rCRS

rCRS: Revised Cambridge Reference Sequence

Participant ID	Age	Base position	Reference Base(rCRS)	Mutant Base	Mutant Frequency	Heteroplasmy	Gene	Amino acid change	Reported Polymorphism
BCC044 BUC	72	150	C	T	72	3.25	Non-Coding		Yes
BCC044 BUC	72	152	T	C	69	3.12	Non-Coding		Yes
BCC044 BUC	72	189	A	G	36	1.43	Non-Coding		Yes
BCC044 BUC	72	204	T	C	22	0.88	Non-Coding		Yes
BCC044 BUC	72	7028	T (Germline Ref)	C	24	0.93	MT-CO1	Silent	Yes
BCC044 BUC	72	14093	T	C	14	0.8	MT-ND5	p.L586P	No
BCC047 BUC	76	234	A	G	283	9.67	Non-Coding		Yes
BCC047 BUC	76	374	A	G	13	0.85	Non-Coding		Yes
BCC098 BUC	17	4340	A	G	17	0.83	MT-TQ		Yes
BCC098 BUC	17	7028	T (Germline Ref)	C	20	0.86	MT-CO1	Silent	Yes
BCC098 BUC	17	12308	G (Germline Ref)	A	49	1.23	MT-TL2		Yes
BCC110 BUC	24	3915	A (Germline Ref)	G	42	1.92	MT-ND1	Silent	Yes
BCC110 BUC	24	4935	A	G	12	0.8	MT-ND2	p.T156A	Yes
BCC110 BUC	24	11253	C (Germline Ref)	T	32	0.86	MT-ND4	Silent	Yes
BCC112 BUC	25	146	T	C	19	0.86	Non-Coding		Yes
BCC112 BUC	25	8994	A (Germline Ref)	G	27	1.07	MT-ATP6	Silent	Yes
BCC112 BUC	25	14053	G (Germline Ref)	A	35	1.99	MT-ND5	Silent	Yes
BCC112 BUC	25	14692	A	G	12	0.81	MT-TE		No
BCC113 BUC	78	185	A (Germline Ref)	G	91	2.67	Non-Coding		Yes
BCC113 BUC	78	189	A	G	59	1.72	Non-Coding		Yes
BCC113 BUC	78	10084	C (Germline Ref)	T	189	3.4	MT-ND3	Silent	Yes
BCC113 BUC	78	11684	T	C	38	0.9	MT-ND4	p.F309L	Yes
BCC113 BUC	78	15757	G (Germline Ref)	A	52	1.48	MT-CYB	Silent	Yes
BCC117 BUC	25	1811	G (Germline Ref)	A	33	1.14	MT-RNR2		Yes
BCC117 BUC	25	3480	G (Germline Ref)	A	12	1.31	MT-ND1	Silent	Yes
BCC117 BUC	25	5539	A	G	11	1.17	MT-TW		Yes
BCC117 BUC	25	7028	T (Germline Ref)	C	11	0.84	MT-CO1	Silent	Yes
BCC117 BUC	25	9698	C (Germline Ref)	T	9	0.9	MT-CO3	Silent	Yes
BCC117 BUC	25	10550	G (Germline Ref)	A	42	0.98	MT-ND4L	Silent	Yes
BCC117 BUC	25	11467	G (Germline Ref)	A	50	1.04	MT-ND4	Silent	Yes
BCC117 BUC	25	12308	G (Germline Ref)	A	58	1.33	MT-TL2		Yes
BCC165 BUC	25	4820	A (Germline Ref)	G	14	0.84	MT-ND2	Silent	Yes
BCC165 BUC	25	12308	G (Germline Ref)	A	47	1.08	MT-TL2		Yes
BCC180 BUC	25	16443	T	C	19	0.81	Non-Coding		No
BCC180 BUC	25	16541	A	G	9	0.87	Non-Coding		No
BCC205 BUC	23	4677	C	T	41	1.89	MT-ND2	p.L70F	No
BCC205 BUC	23	5973	G	A	94	4.4	MT-CO1	p.A24T	Yes
BCC205 BUC	23	13094	T	C	16	0.83	MT-ND5	p.V253A	No

Participant ID	Age	Base position	Reference Base(rCRS)	Mutant Base	Mutant Frequency	Heteroplasmy	Gene	Amino acid change	Reported Polymorphism
BCC213 BUC	71	152	T	C	23	0.94	Non-Coding		Yes
BCC213 BUC	71	3424	G	A	26	0.83	MT-ND1	p.V40M	No
BCC213 BUC	71	14858	G	A	63	4.12	MT-CYB	p.G38S	Yes
BCC213 BUC	71	15456	T	C	16	0.87	MT-CYB	p.L237P	No
BCC222BUC	75	146	T	C	75	1.65	Non-Coding		Yes
BCC222BUC	75	189	A	G	39	0.8	Non-Coding		Yes
BCC222BUC	75	215	A	G	38	0.85	Non-Coding		Yes
BCC244 BUC	23	295	T (Germline Ref)	C	20	1.15	Non-Coding		Yes
BCC244 BUC	23	3991	A	G	18	0.81	MT-ND1	p.T229A	No
BCC244 BUC	23	7028	T (Germline Ref)	C	21	0.81	MT-CO1	Silent	Yes
BCC244 BUC	23	9633	T	C	13	0.91	MT-CO3	p.S143P	Yes
BCC260 BUC	71	234	A	G	75	4.03	Non-Coding		Yes
BCC260 BUC	71	7028	T (Germline Ref)	C	23	0.82	MT-CO1	Silent	Yes
BCC260 BUC	71	9029	A	G	18	0.93	MT-ATP6	p.H168R	Yes
BCC260 BUC	71	12007	A (Germline Ref)	G	34	1.37	MT-ND4	Silent	Yes
BCC260 BUC	71	13969	A	G	16	0.98	MT-ND5	p.S545G	No
BCC260 BUC	71	15281	T	C	16	0.85	MT-CYB	p.F179L	No
BCC260 BUC	71	16129	A (Germline Ref)	G	41	1.48	Non-Coding		Yes
BCC260 BUC	71	16390	A (Germline Ref)	G	22	1.09	Non-Coding		Yes
BCC260 BUC	71	16486	A	G	12	0.85	Non-Coding		No
BCC265 BUC	73	204	T	C	9	0.9	Non-Coding		Yes
BCC265 BUC	73	6371	T (Germline Ref)	C	26	1.15	MT-CO1	Silent	Yes
BCC265 BUC	73	7028	T (Germline Ref)	C	21	0.82	MT-CO1	Silent	Yes
BCC265 BUC	73	15454	T	C	16	0.83	MT-CYB	Silent	Yes
BCC265 BUC	73	16223	T (Germline Ref)	C	12	1.03	Non-Coding		Yes
BCC266 BUC	74	7028	T (Germline Ref)	C	24	0.89	MT-CO1	Silent	Yes
BCC266 BUC	74	12880	T	C	22	0.8	MT-ND5	p.F182L	Yes
BCC266 BUC	74	13935	A	G	16	0.81	MT-ND5	Silent	No
BCC266 BUC	74	16290	C	T	10	0.87	Non-Coding		Yes
