# Supplementary material for: Clonal Expansion of Early to Mid-Life Mitochondrial DNA Point Mutations Drives Mitochondrial Dysfunction during Human Ageing
Source: PLoS Genet. 2014 Sep 18;10(9):e1004620. doi: 10.1371/journal.pgen.1004620 (PMC4169240; doi:10.1371/journal.pgen.1004620)
Supplement: Table S4 — Validation of the sensitivity and specificity of the RMC assay. (PDF) [file pgen.1004620.s005.pdf]

Expected number of mutant copies per PCR reaction	Expected mutation frequency	Average observed mutation frequency (n=3)
10	$4x10^{-3}$	$4.00x10^{-3} \pm 0$
1	$4x10^{-4}$	$3.69x10^{-4} \pm 4.8 x10^{-5}$
0.1	$4x10^{-5}$	$4.27x10^{-5} \pm 2.0x10^{-5}$
0.01	$4x10^{-6}$	$4.33x10^{-6} \pm 5.13x10^{-6}$

Table S4: Validation of the sensitivity and specificity of the RMC assay. Wild-type and mutant clones were mixed in known concentrations and the RMC assay carried out. This was done on 3 separate occasions. The observed mtDNA mutation frequencies were compared to the expected mtDNA mutation frequencies. There was no significant difference between the observed and expected frequencies ($p=0.99$ chi-squared test).
