# Supplementary material for: Clonal Expansion of Early to Mid-Life Mitochondrial DNA Point Mutations Drives Mitochondrial Dysfunction during Human Ageing
Source: PLoS Genet. 2014 Sep 18;10(9):e1004620. doi: 10.1371/journal.pgen.1004620 (PMC4169240; doi:10.1371/journal.pgen.1004620)
Supplement: Table S5 — MtDNA variants detected in the cloned mtDNA plasmid control. (PDF) [file pgen.1004620.s006.pdf]

Table S5: MtDNA variants detected in the cloned mtDNA plasmid control.

The variants highlighted in red were also detected in all of the colon and buccal samples and were therefore excluded from the analysis in all cases

No variants were detected >0.64%.

rCRS: Revised Cambridge Reference Sequence

Base Position	Reference Base (rCRS)	Base Change	Coverage	Quality Score	Variant Frequency	Variant Heteroplasmy
108	A	G	52700	27.5	161	0.31
152	C (Germline Ref)	T	61695	35.9	87	0.14
172	T	C	61993	27.7	192	0.31
175	A	G	61923	36.4	193	0.31
183	A	G	60372	22.3	194	0.32
223	T	C	46791	31	162	0.35
263	G (Germline Ref)	A	36164	34.9	67	0.19
270	A	G	34129	26.9	118	0.35
272	A	G	33177	24.8	110	0.33
567	A	G	12588	24.4	49	0.39
588	T	C	17555	22.7	54	0.31
608	A	G	24101	31.2	77	0.32
659	T	C	38321	20.3	129	0.34
678	T	C	47106	28.4	144	0.31
721	T	C	59507	29.1	227	0.38
723	A	G	59968	21.1	192	0.32
735	A	G	62042	34.8	199	0.32
750	G (Germline Ref)	A	66481	31.2	852	1.28
753	A	G	65823	31.4	251	0.38
758	T	C	65818	25.7	231	0.35
760	A	G	65797	31.5	224	0.34
776	A	G	65882	31.3	279	0.42
819	A	G	65796	30	225	0.34
827	A	G	65943	24.1	252	0.38
879	T	C	65990	24.6	360	0.55
888	T	C	65834	29.8	273	0.41
905	A	G	65760	24.7	205	0.31
907	A	G	65813	29.3	255	0.39

Base Position	Reference Base (rCRS)	Base Change	Coverage	Quality Score	Variant Frequency	Variant Heteroplasmy
921	T	C	65815	33.1	247	0.38
929	A	G	65929	28.1	256	0.39
942	A	G	65827	33	276	0.42
980	T	C	44741	23.5	167	0.37
1010	A	G	60464	23.8	233	0.39
1019	A	G	62734	29.2	219	0.35
1033	T	C	65751	32.4	203	0.31
1051	A	G	65841	36.9	285	0.43
1063	A	G	65787	31.2	208	0.32
1101	T	C	65807	28.3	215	0.33
1107	T	C	65919	22.7	328	0.5
1132	T	C	65809	23.4	233	0.35
1139	A	G	65796	33.4	245	0.37
1142	A	G	65852	30.5	287	0.44
1164	T	C	65826	25.1	264	0.4
1229	T	C	65810	26.2	232	0.35
1237	A	G	65764	34.3	213	0.32
1269	T	C	65810	26.2	257	0.39
1272	A	G	65769	24.8	203	0.31
1286	A	G	65793	25.6	237	0.36
1287	A	G	66656	25.7	257	0.39
1306	A	G	65878	32.8	251	0.38
1329	T	C	65894	24.7	290	0.44
1338	A	G	65819	28.9	231	0.35
1349	T	C	65861	26.9	281	0.43
1384	A	G	67571	28.8	232	0.34
1423	A	G	65790	27.2	237	0.36
1446	A	G	65925	31.6	337	0.51
1492	A	G	65755	24	203	0.31
1509	T	C	65775	33.7	217	0.33
1530	A	G	64139	32.7	193	0.3
1555	A	G	59938	24.9	215	0.36
1557	A	G	59958	30.9	180	0.3
1587	T	C	52702	22.6	190	0.36

Base Position	Reference Base (rCRS)	Base Change	Coverage	Quality Score	Variant Frequency	Variant Heteroplasmy
1590	A	G	51662	33.2	223	0.43
1670	A	G	31965	33.8	153	0.48
1761	A	G	31368	32.4	96	0.31
1777	A	G	34125	26.9	123	0.36
1887	A	G	56687	28.9	250	0.44
1900	A	G	58928	33.5	238	0.4
1914	A	G	63445	32.7	265	0.42
1917	A	G	63067	34.2	200	0.32
1931	A	G	65832	25.9	222	0.34
1940	A	G	65815	21.6	257	0.39
1986	A	G	65820	31	256	0.39
1991	A	G	65790	35.6	234	0.36
2035	T	C	65796	27.3	218	0.33
2106	A	G	61596	31.4	189	0.31
2109	A	T	60863	28.9	162	0.27
2110	A	AT	60647	29.6	223	0.37
2112	A	G	60310	30.9	204	0.34
2115	T	C	60438	29.8	232	0.38
2148	A	G	52878	23.5	242	0.46
2184	A	G	46422	20.5	153	0.33
2208	A	G	43318	31.2	137	0.32
2214	A	G	42531	29.3	138	0.32
2216	A	G	42357	29	158	0.37
2260	A	G	41359	23.3	185	0.45
2270	A	G	41833	21.7	150	0.36
2327	T	C	43227	21	155	0.36
2365	T	C	43328	29	165	0.38
2367	A	G	43298	34.1	164	0.38
2369	A	G	43637	31.2	145	0.33
2422	T	C	50469	27	218	0.43
2424	A	G	51254	33.1	174	0.34
2432	A	G	52949	33.2	179	0.34
2442	T	C	53658	28.3	228	0.42
2510	T	C	65829	22.8	228	0.35

Base Position	Reference Base (rCRS)	Base Change	Coverage	Quality Score	Variant Frequency	Variant Heteroplasmy
2512	A	C	65832	32.9	276	0.42
2515	T	C	65797	32.7	218	0.33
2552	T	C	65780	32.3	228	0.35
2554	A	G	65994	29	341	0.52
2609	T	C	65786	28	236	0.36
2632	A	G	65808	29.1	235	0.36
2649	T	C	65931	24.2	248	0.38
2651	A	G	65982	29.5	363	0.55
2654	T	C	66002	30.9	397	0.6
2656	T	C	66034	29.8	383	0.58
2658	T	C	65934	29.8	343	0.52
2660	T	C	65901	29.9	327	0.5
2664	T	C	65904	28.8	319	0.48
2674	T	C	65763	30.9	211	0.32
2682	A	G	66061	28.4	423	0.64
2685	T	C	65869	20.5	244	0.37
2717	A	G	65951	30.8	346	0.52
2720	A	G	65792	20.3	237	0.36
2722	A	G	65798	29.2	242	0.37
2723	A	G	65961	23	258	0.39
2725	A	G	65905	28.2	330	0.5
2737	T	C	65819	30.3	253	0.38
2775	A	G	65935	23.8	296	0.45
2830	A	G	65931	21.3	273	0.41
2833	A	G	65896	25.9	222	0.34
2854	T	C	65823	33	244	0.37
2863	T	C	65880	23.8	299	0.45
2864	T	C	66692	32.2	260	0.39
2866	A	G	65814	24.8	252	0.38
2879	A	G	65759	33.5	204	0.31
2880	A	G	65918	32.1	273	0.41
2885	T	C	65794	21.8	221	0.34
2910	A	G	65911	24.8	274	0.42
2921	A	G	65898	31.3	329	0.5

Base Position	Reference Base (rCRS)	Base Change	Coverage	Quality Score	Variant Frequency	Variant Heteroplasmy
2922	A	G	65887	24.6	223	0.34
2924	T	C	65817	30.5	259	0.39
2938	A	G	65955	25.9	268	0.41
2940	A	G	65831	23.3	235	0.36
2947	T	C	65854	28.6	270	0.41
2950	T	C	65769	27.8	198	0.3
2960	T	C	65926	28.7	297	0.45
2987	T	C	65902	20.5	320	0.49
3000	A	G	65962	27	336	0.51
3006	T	C	65827	23.5	239	0.36
3010	A	G	65887	35.5	164	0.25
3018	A	G	66065	29	423	0.64
3037	T	C	65762	29.9	202	0.31
3041	T	C	65836	34.7	292	0.44
3045	A	G	65871	23.9	204	0.31
3052	A	G	65839	33.2	230	0.35
3055	T	C	65967	20.1	257	0.39
3062	T	C	65928	34.3	362	0.55
3065	T	C	65816	26.3	243	0.37
3067	T	C	65898	20.7	310	0.47
3069	A	G	65839	32.1	279	0.42
3102	T	C	65829	33.4	262	0.4
3133	A	G	65797	34.4	227	0.35
3135	A	G	65845	36.7	280	0.43
3136	A	G	65903	20.8	218	0.33
3138	A	G	65826	34.7	268	0.41
3282	A	G	36984	20.9	116	0.31
3304	A	G	37152	25.6	113	0.3
3611	T	C	12861	24.6	39	0.3
3628	A	G	17192	28.5	52	0.3
3669	A	G	26930	23.3	109	0.4
3708	A	G	36464	22	121	0.33
3760	T	C	46362	34.3	150	0.32
3805	A	G	50654	23.7	164	0.32

Base Position	Reference Base (rCRS)	Base Change	Coverage	Quality Score	Variant Frequency	Variant Heteroplasmy
3818	A	G	50477	29.7	169	0.33
3883	A	G	47101	28	160	0.34
3902	A	G	45768	34.7	622	1.36
3904	C	A	46054	34.7	548	1.19
3906	T	G	46076	30	92	0.2
3908	C	T	46430	34.7	630	1.36
3917	A	G	45522	35.4	173	0.38
3929	T	C	46342	21.5	194	0.42
3931	T	C	46661	26.2	156	0.33
3937	T	C	46539	31.7	144	0.31
3991	A	G	37500	27.5	157	0.42
4049	A	G	23729	30.8	104	0.44
4072	A	G	23608	30.4	86	0.36
4087	A	G	22706	31.7	91	0.4
4154	A	G	20861	21.7	71	0.34
4166	A	G	22498	25.8	68	0.3
4220	T	C	28860	24.6	94	0.33
4283	A	G	41919	20.6	131	0.31
4297	A	G	43340	27.5	131	0.3
4340	A	G	42763	34.4	166	0.39
4388	A	G	47737	21.1	209	0.44
4520	A	G	45636	26	184	0.4
4525	T	C	46306	28.5	184	0.4
4527	A	G	46870	26.9	159	0.34
4543	A	G	47545	32.2	158	0.33
4557	T	C	47959	34.8	147	0.31
4586	T	C	44854	29.7	191	0.43
4622	T	C	47381	30	157	0.33
4653	A	G	49383	23.6	149	0.3
4721	A	G	47674	33.7	154	0.32
4801	T	C	48275	36.3	154	0.32
4866	T	C	41338	24.8	125	0.3
5376	A	G	7460	28.2	25	0.34
5474	A	G	11533	23.8	37	0.32

Base Position	Reference Base (rCRS)	Base Change	Coverage	Quality Score	Variant Frequency	Variant Heteroplasmy
5539	A	G	32270	32.4	133	0.41
5589	A	G	45457	30.1	153	0.34
5594	T	C	47276	28	191	0.4
5630	A	G	57776	28.5	214	0.37
5732	T	C	65996	30.5	317	0.48
5734	T	C	65911	32.8	305	0.46
5785	T	C	56967	36.8	218	0.38
5814	T	C	57777	28.2	190	0.33
5816	A	G	57782	35	202	0.35
5836	A	G	56841	32	226	0.4
5848	T	C	56349	31.6	225	0.4
5850	T	C	56416	34.2	178	0.32
5873	T	C	54105	35.2	183	0.34
5904	A	G	54705	33.7	175	0.32
5907	T	C	54666	34.8	177	0.32
5921	A	G	52742	32.1	214	0.41
5944	A	G	55143	30.7	186	0.34
5978	A	G	57319	30.4	250	0.44
6040	G (Germline Ref)	A	66265	37.8	159	0.24
6070	T	C	65760	31.1	205	0.31
6076	T	C	65926	34.9	343	0.52
6080	A	G	65878	28.3	287	0.44
6086	T	C	65810	28.9	243	0.37
6100	T	C	65823	24.8	245	0.37
6102	T	C	65755	29	207	0.31
6121	T	C	65932	31.2	327	0.5
6141	A	G	65772	33.4	218	0.33
6144	T	C	65776	32.3	202	0.31
6146	A	G	65889	33.9	293	0.44
6167	T	C	65762	23.5	199	0.3
6183	T	C	65903	24.1	324	0.49
6184	T	C	65778	37.2	228	0.35
6204	A	G	65894	21.2	210	0.32
6210	T	C	65872	29.8	276	0.42

Base Position	Reference Base (rCRS)	Base Change	Coverage	Quality Score	Variant Frequency	Variant Heteroplasmy
6214	T	C	65813	33.1	261	0.4
6229	T	C	65870	26.2	259	0.39
6235	T	C	65862	20.8	255	0.39
6241	T	C	65816	29.4	247	0.38
6256	T	C	65777	30.4	224	0.34
6259	A	G	65781	22.1	213	0.32
6266	A	G	65759	31.7	203	0.31
6269	A	G	65872	28.6	266	0.4
6345	T	C	32087	29.2	113	0.35
6367	T	C	31547	26.4	129	0.41
6459	T	C	24463	34.6	75	0.31
6481	T	C	28139	30.6	94	0.33
6495	T	C	29903	31.1	100	0.33
6565	A	G	34303	20.7	150	0.44
6583	A	G	42656	27.9	179	0.42
6698	A	G	64496	33	235	0.36
6700	A	G	64028	34.6	234	0.37
6728	A	G	65783	31	220	0.33
6763	T	C	65777	35.2	230	0.35
6767	A	G	65948	22.8	356	0.54
6772	A	G	65806	30.5	256	0.39
6796	A	G	65851	34.1	277	0.42
6802	A	G	65918	31.9	307	0.47
6815	T	C	65757	33.7	211	0.32
6879	A	G	65849	28.8	279	0.42
6881	A	G	65829	32.3	252	0.38
6919	T	C	65822	27	238	0.36
6937	T	C	65814	33.1	226	0.34
6939	T	C	65872	34.3	301	0.46
6989	A	G	65758	30	206	0.31
7006	A	G	65777	25.9	225	0.34
7032	T	C	65266	35	228	0.35
7058	T	C	60594	31.8	268	0.44
7150	T	C	61205	28.1	188	0.31

Base Position	Reference Base (rCRS)	Base Change	Coverage	Quality Score	Variant Frequency	Variant Heteroplasmy
7175	T	C	56128	27.4	181	0.32
7189	A	G	55880	31.1	195	0.35
7263	T	C	64703	28.5	230	0.36
7275	T	C	64554	33.5	215	0.33
7286	T	C	65798	31.3	239	0.36
7324	A	G	63743	32.6	246	0.39
7344	A	G	40496	27.2	128	0.32
7445	A	G	31340	33.8	114	0.36
7480	T	C	41807	29.9	158	0.38
7507	A	G	49198	34.5	159	0.32
7545	T	C	65779	35.1	229	0.35
7594	T	C	65886	31.4	283	0.43
7597	A	G	65914	24.7	289	0.44
7617	A	G	65816	29	238	0.36
7848	A	G	24224	30.6	89	0.37
7856	A	G	27043	27.6	106	0.39
7901	T	C	40230	29.1	139	0.35
7980	A	G	62344	28.7	212	0.34
7983	T	C	63564	25.6	221	0.35
7987	A	G	64003	29.3	224	0.35
8001	A	G	65888	31.4	281	0.43
8008	A	G	65859	23.6	251	0.38
8025	A	G	65797	21.8	249	0.38
8058	A	G	65924	27.1	297	0.45
8067	A	G	65831	28.7	274	0.42
8069	T	C	65852	36.2	298	0.45
8074	A	G	65858	32.9	280	0.43
8077	T	C	65946	23.1	314	0.48
8103	A	G	65822	27.5	258	0.39
8146	A	G	65868	23.6	300	0.46
8200	T	C	65865	35.9	294	0.45
8531	A	G	12891	22.5	50	0.39
8653	A	G	10377	27.7	40	0.39
8725	A	G	17113	27.8	64	0.37

Base Position	Reference Base (rCRS)	Base Change	Coverage	Quality Score	Variant Frequency	Variant Heteroplasmy
8733	T	C	17907	23.4	60	0.34
8795	A	G	18803	21.3	72	0.38
8860	G (Germline Ref)	A	17900	32.2	24	0.13
8875	T	C	16807	29	70	0.42
8979	T	C	4278	26.4	14	0.33
9041	A	G	11252	31.4	42	0.37
9052	A	G	13078	25.1	58	0.44
9098	T	C	20829	25.9	65	0.31
9306	T	C	54349	34	166	0.31
9315	T	C	55203	34.5	171	0.31
9404	A	G	59970	26.2	210	0.35
9420	A	G	54840	34.9	206	0.38
9422	A	G	53787	29.8	185	0.34
9503	A	G	44692	31.6	150	0.34
9745	A	G	65145	32.7	210	0.32
9758	T	C	65781	29.4	220	0.33
9789	T	C	65772	32.4	203	0.31
9813	T	C	65883	35.7	321	0.49
9823	T	C	65780	35	222	0.34
9840	T	C	65891	30.4	303	0.46
9845	T	C	65847	34.5	280	0.43
9856	T	C	65873	28.1	286	0.43
9861	T	C	65821	33.9	269	0.41
9879	T	C	65796	31.3	240	0.36
9880	T	C	65768	36.6	204	0.31
9885	T	C	65796	32.4	232	0.35
9899	T	C	65898	25.9	313	0.47
9909	T	C	65851	28	294	0.45
9930	T	C	65889	25	293	0.44
9944	T	C	65818	31.4	254	0.39
9946	T	C	65790	28.6	227	0.35
9953	A	G	65849	22.3	237	0.36
9961	T	C	65778	24.1	206	0.31
9967	T	C	65921	30.9	309	0.47

Base Position	Reference Base (rCRS)	Base Change	Coverage	Quality Score	Variant Frequency	Variant Heteroplasmy
9969	T	C	65850	24	245	0.37
9989	T	C	65812	21.1	229	0.35
10089	A	G	49771	22.5	189	0.38
10376	A	G	52671	26.5	190	0.36
10468	A	C	65880	23.6	68	0.36
10510	T	C	63623	25.9	274	0.43
10665	T	C	31531	25.9	128	0.41
10682	A	G	33262	22.5	116	0.35
10705	T	C	33837	25.9	111	0.33
10802	A	G	26122	28.3	89	0.34
10804	A	G	25979	27.9	94	0.36
10807	A	G	25678	24.3	87	0.34
10821	A	G	23403	31.8	75	0.32
11118	T	C	36732	28.4	132	0.36
11267	A	G	29291	32.5	117	0.4
11299	T	C	33791	30.8	111	0.33
11318	T	C	36130	21.2	115	0.32
11329	A	G	37198	29.7	119	0.32
11357	A	G	39442	31.9	140	0.35
11500	A	G	34684	31.6	117	0.34
11526	A	G	25114	23	95	0.38
11579	T	C	31602	31	102	0.32
11601	A	G	35260	32.7	138	0.39
11624	T	C	37562	27.1	113	0.3
11678	T	C	43939	34.1	136	0.31
11781	T	C	52664	31.3	163	0.31
11809	T	C	50765	21.4	167	0.33
12114	A	G	50691	23.4	164	0.32
12177	A	G	61837	20.8	205	0.33
12184	A	G	62231	22.2	218	0.35
12215	T	C	63725	32	235	0.37
12217	A	G	64112	28.8	195	0.3
12232	T	C	62825	34.1	211	0.34
12317	T	C	31935	23.3	140	0.44

Base Position	Reference Base (rCRS)	Base Change	Coverage	Quality Score	Variant Frequency	Variant Heteroplasmy
12347	A	G	20286	28.3	61	0.3
12473	T	C	19866	23.7	61	0.31
12479	T	C	20256	27.7	77	0.38
12497	T	C	22864	26.9	81	0.35
12500	T	C	23290	25.2	71	0.3
12534	A	G	26836	31.2	118	0.44
12536	A	G	26965	20.5	110	0.41
12540	A	G	27355	32	123	0.45
12631	T	C	51167	31.3	174	0.34
12654	A	G	56477	30.1	183	0.32
12757	T	C	65857	33.7	307	0.47
12766	T	C	65798	27.5	223	0.34
12793	T	C	65786	28.9	223	0.34
12807	A	G	65824	34.2	243	0.37
12810	A	G	65814	24.1	211	0.32
12834	A	G	65778	26.3	200	0.3
12924	A	G	46903	32.2	165	0.35
13057	A	G	23521	30.8	87	0.37
13254	T	C	37953	27.9	123	0.32
13260	T	C	37466	27.5	122	0.33
13262	A	G	37605	34.7	119	0.32
13304	A	G	34880	28.4	117	0.34
13363	T	C	39453	27.9	144	0.36
13373	T	C	39040	21.4	122	0.31
13441	A	G	36839	31.8	118	0.32
13540	T	C	24359	22.8	82	0.34
13572	T	C	23484	20.4	76	0.32
13591	A	G	23182	27.3	82	0.35
13799	T	C	15298	28.5	53	0.35
13812	T	C	17259	22.7	52	0.3
13814	T	C	17340	24.1	55	0.32
14007	A	G	11095	27.3	35	0.32
14065	A	G	12998	26	41	0.32
14093	T	C	13889	26.3	51	0.37

Base Position	Reference Base (rCRS)	Base Change	Coverage	Quality Score	Variant Frequency	Variant Heteroplasmy
14110	T	C	14545	21.7	45	0.31
14679	T	C	10920	25.6	41	0.38
14692	A	G	11440	26.3	50	0.44
14723	T	C	12370	30.1	43	0.35
14743	A	G	10761	22.9	36	0.33
14851	A	G	17188	24.1	60	0.35
14853	T	C	18115	31	60	0.33
14919	A	G	35491	23.5	116	0.33
14939	T	C	43442	33.4	142	0.33
14961	A	G	49703	24.3	211	0.42
15023	T	C	65804	22.9	229	0.35
15069	T	C	65784	26.4	226	0.34
15074	T	C	65765	31.1	217	0.33
15099	T	C	65777	20.1	213	0.32
15161	T	C	65822	34.7	247	0.38
15178	A	G	65839	27	248	0.38
15204	T	C	65791	21.8	202	0.31
15220	A	G	65767	21.7	205	0.31
15222	A	G	65802	29.5	232	0.35
15293	T	C	35731	32.4	124	0.35
15299	T	C	34482	27.6	109	0.32
15325	A	G	27765	30.6	133	0.48
15326	G (Germline Ref)	A	27077	33.4	34	0.13
15344	T	C	24802	27.2	84	0.34
15766	A	G	62054	31.5	198	0.32
15768	A	G	62806	27.3	226	0.36
15852	T	C	65817	27.1	251	0.38
15889	T	C	65807	31.1	228	0.35
15911	A	G	65766	35	215	0.33
15916	T	C	65842	34.7	267	0.41
15918	T	C	65856	32	278	0.42
15934	A	G	65817	30.3	232	0.35
15935	A	G	65786	37	233	0.35
15936	A	G	65766	36.3	203	0.31

Base Position	Reference Base (rCRS)	Base Change	Coverage	Quality Score	Variant Frequency	Variant Heteroplasmy
15958	A	G	65879	31.1	276	0.42
15960	A	G	65831	29.2	266	0.4
15976	T	C	65789	32.4	223	0.34
15999	A	G	65832	32.6	216	0.33
16017	T	C	65784	27.3	238	0.36
16020	T	C	65847	31.1	286	0.43
16025	T	C	65900	28.9	258	0.39
16027	T	C	65834	28.9	275	0.42
16031	A	G	65773	24.6	200	0.3
16075	T	C	65786	24.9	199	0.3
16105	T	C	56501	31.5	174	0.31
16486	A	G	39775	25.1	131	0.33
16487	A	G	39644	26.8	135	0.34
16519	C (Germline Ref)	T	29217	28.6	45	0.15
